# Lignocellulose degradation characteristics and mechanisms of raw and NaOH-pretreated wheat straw by *Irpex lacteus* QJ: multi-omics analysis

**DOI:** 10.1186/s40643-026-01091-8

**Published:** 2026-07-10

**Authors:** Qijun Zhu, Baorui Liu, Zhengrong Dou, Yunsheng Han, Hongying Cai, Peilong Yang, Weiwei Liu, Kun Meng

**Affiliations:** Institute of Feed Research, Chinese Academy of Agricultural Sciences, Beijing, 100000 China

**Keywords:** *Irpex lacteus* QJ, Wheat straw, NaOH pretreatment, Lignocellulose degradation, Transcriptomic analysis, Metabolomic analysis

## Abstract

The recalcitrant structure of lignocellulose hinders the efficient valorization of wheat straw. White-rot fungi have been extensively studied for their ability to completely degrade lignocellulose, yet the specific mechanisms remain insufficiently elucidated. In this study, the white-rot fungus *Irpex lacteus* QJ was applied to the solid-state fermentation of raw wheat straw (IW group) and NaOH-pretreated wheat straw (IN group). The degradation efficiency and mechanisms were investigated using enzyme activity assays, structural analyses (scanning electron microscopy, Fourier transform infrared spectroscopy, and X-ray diffraction), and multi-omics approaches. The results demonstrated that *I. lacteus* QJ effectively degraded wheat straw in both groups, and the IW group had significantly higher degradation rates of cellulose (56.21% vs. 41.90%), hemicellulose (57.30% vs. 42.94%) and lignin (54.90% vs. 42.23%), and significantly increased contents of crude protein (12.96% vs. 7.07%), thereby reducing the sugar concentration (33.18 mg/g vs. 9.63 mg/g) and lignocellulolytic enzyme activity. The results of the transcriptomic analysis revealed that the pathways enriched by the differentially expressed genes in the two groups were related to lignocellulose degradation. Key enzymes (including phenol hydroxylase, aldehyde dehydrogenase, and D-xylulose reductase) were identified by the protein‒protein interaction network. Metabolomic analysis revealed that the changes in most lignin-related metabolites were consistent between the two groups, and the levels of common metabolites such as methyl cinnamate and 4-acetamidobenzoic acid were strongly positively correlated with the lignocellulose degradation efficiency. In conclusion, this study systematically assessed the lignocellulose degradation performance of *I. lacteus* QJ and clarified its degradation mechanisms via multi-omics analyses, providing a theoretical reference for the fungus-mediated bioconversion of lignocellulosic biomass.

**Graphical abstract:**

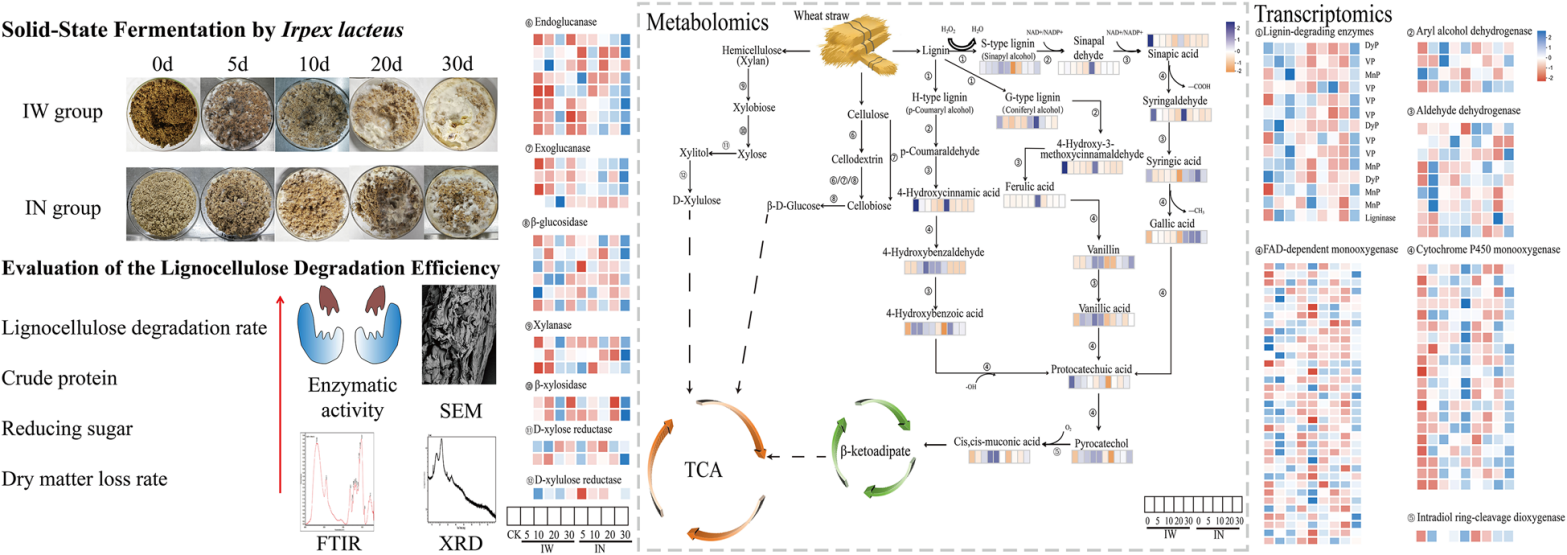

**Supplementary Information:**

The online version contains supplementary material available at 10.1186/s40643-026-01091-8.

## Introduction

Wheat straw (WS), usually being treated as an agricultural waste, is one of the most abundant renewable feedstocks as a typical lignocellulosic biomass (Iram et al. 2021). WS has been used for various purposes, such as animal feed, fertilizer, and clean fuel (Akbari Moghaddam Kakhki et al. 2024; Dashora et al. 2022; Yang et al. 2024; Yue et al. 2025). However, the complex structure of lignocellulose, particularly the recalcitrant three-dimensional network formed by lignin, the high crystallinity of cellulose, and the covalent bonds between lignin and cellulose/hemicellulose, severely hinders its degradation and effective utilization (Chen et al. 2025). For instance, when used as feed, owing to its complex structure, this lignocellulosic biomass resists degradation by intestinal enzymes and microorganisms in livestock, which limits its application and significantly increases costs (Moscariello et al. 2024). Currently, the main pretreatment methods used to improve the utilization efficiency of lignocellulose include physical, chemical, and biological approaches. Notably, biological methods have attracted considerable attention because of their mild reaction conditions, environmental friendliness, low cost, and ability to improve nutritional content.

Fungi are capable of fully degrading lignocellulose under natural conditions. White-rot fungi, in particular, can completely mineralize recalcitrant lignin into CO_2_ and water. This process disrupts the composite structure of lignocellulose, releasing cellulose and hemicellulose and facilitating the activity of cellulase and hemicellulase (Datsomor et al. 2022; Yang and Hu 2025). Among them, *Irpex lacteus* is widely used in straw treatment because of its strong substrate adaptability and high degradation efficiency (Niu et al. 2025, 2020). Niu et al. (2018) demonstrated that compared with *Pleurotus ostreatus* and *Phanerochaete chrysosporium*, *I. lacteus* exhibits superior lignin degradation and has the ability to degrade cellulose and hemicellulose to a moderate extent. Furthermore, the crude protein content of corn stover significantly increases after fermentation with *I. lacteus*, exceeding the levels achieved with *P. ostreatus* and *Pleurotus cystidiosus* (Zuo et al. 2018). Additionally, the release of xylose and glucose from WS after *I. lacteus* fermentation pretreatment is markedly greater than that of *Ceriporiopsis subvermispora* (Salvachúa et al. 2013; Wan and Li 2011). Although *I. lacteus* has displayed excellent lignocellulose bioconversion capabilities, research on the mechanisms underlying its degradation potential remains limited.

Advances in multi-omics technologies have greatly facilitated the identification of the potential genes, proteins, metabolites, and pathways involved in lignocellulose degradation. For instance, combined transcriptomic and metabolomic analyses have revealed that compared with other carbon sources, *Ruminiclostridium papyrosolvens* exhibits more extensive gene expression and more abundant metabolites when growing on corn stover as a carbon source and that it degrades lignocellulose via the CAZymes-ABC transporter regulatory network (You et al. 2025). In-depth investigations of these genes and metabolites not only deepen our understanding of microbial lignocellulose degradation mechanisms but also provide a theoretical basis for improving lignocellulose bioconversion efficiency. However, studies that use integrated multi-omics approaches to explore the mechanisms through which *I. lacteus* degrades lignocellulose are relatively rare.

In this study, the mechanism through which *I. lacteus* QJ degrades WS was investigated using morphological, biochemical, transcriptomic, and metabolomic approaches. Solid-state fermentation (SSF) experiments confirmed that the *I. lacteus* QJ isolated by our team is capable of efficiently degrading WS lignocellulose. Enzyme activity assays demonstrated that *I. lacteus* QJ possesses a complete and highly efficient lignocellulolytic enzyme system. Scanning electron microscopy, infrared spectroscopy, and X-ray diffraction were employed to examine changes in WS morphology, lignocellulosic functional groups, and cellulose crystallinity during degradation by *I. lacteus* QJ. Additionally, we conducted integrated transcriptomic and metabolomic analyses to systematically explore the potential mechanisms underlying efficient lignocellulose degradation by *I. lacteus* QJ, identifying the key enzymes, metabolites, and regulatory pathways. This research provides a theoretical basis for the application of *I. lacteus* in WS utilization.

## Materials and methods

### Fungal strain preparation

The white-rot fungus *I. lacteus* QJ was originally isolated by our team from rotten straw at the Chinese Academy of Agricultural Sciences (Beijing, China) and deposited at the China General Microbiological Culture Collection Center (Accession No. CGMCC 41264). The strain was first cultured on potato dextrose agar (PDA) plates at 30 °C for 5 d. Mycelial blocks were then inoculated into potato dextrose broth (PDB) and incubated in a shaker at 30 °C and 200 rpm for 5 d. The resulting mycelium was subsequently used for SSF.

### Preparation of WS and SSF

The WS used in this study was collected from the experimental farm of the Chinese Academy of Agricultural Sciences. It was crushed, sieved (20–40 mesh), dried at 65 °C to a constant weight, and then sealed for storage. For pretreatment, WS powder was soaked in a 3% sodium hydroxide solution at room temperature for 24 h, rinsed with running water until it reached a neutral pH, and dried again to obtain NaOH-pretreated WS. For SSF, raw WS and NaOH-pretreated WS were mixed separately with the culture medium (containing 2.5 g/L KH_2_PO_4_, 2.5 g/L MgSO_4_, 15 g/L (NH_4_)_2_SO_4_, and 25 g/L glucose) at a mass ratio of 1:2, followed by sterilization at 115 °C for 20 min. Inoculation was performed using a cylindrical 40-mesh sieve (5 cm diameter, 2 cm height) to control the inoculum volume to 50% of the sieve capacity, followed by incubation at 30 °C. The raw WS group was designated as the IW group and the NaOH-pretreated WS group as the IN group. Samples were collected on Days 5, 10, 20, and 30 to determine the contents of lignocellulose, dry matter, crude protein, and reducing sugars, using Day 0 as the control. Enzyme activities were measured every 5 days. Each treatment was analyzed in three biological replicates at each time point.

### Determination of lignocellulose, crude protein, and reducing sugar contents

Neutral detergent fiber (NDF), acid detergent fiber (ADF), acid detergent lignin (ADL), and crude fiber (CF) were determined by the Van Soest method (Van Soest et al. 1991). The cellulose content was calculated as ADF minus ADL, the hemicellulose content was calculated as NDF minus ADF, and the lignin content was calculated as ADL. The degradation rate was calculated using the following formula:

$$\mathrm{D}\mathrm{e}\mathrm{g}\mathrm{r}\mathrm{a}\mathrm{d}\mathrm{a}\mathrm{t}\mathrm{i}\mathrm{o}\mathrm{n} \mathrm{r}\mathrm{a}\mathrm{t}\mathrm{e} \left(\mathrm{\%}\right)= \frac{{\mathrm{m}}_{1}*{\mathrm{x}}_{1}-{\mathrm{m}}_{2}*{\mathrm{x}}_{2}}{{\mathrm{m}}_{1}*{\mathrm{x}}_{1}}*100\mathrm{\%}$$

where m_1_ and x_1_ denote the dry matter weight and lignocellulose content of WS on Day 0, respectively, and m_2_ and x_2_ represent the corresponding values at each fermentation time point, respectively. The crude protein content was determined using a Dumas nitrogen analyzer (Saint-Denis and Goupy 2004). Reducing sugars were measured using the 3,5-dinitrosalicylic acid (DNS) method (Miller 1959).

### Collection of crude enzyme solution and determination of lignocellulose enzyme activity

Every 5 days, 2 g of fresh fermented WS was collected for enzyme activity analysis and an equivalent sample was dried for dry matter determination. Fresh samples were suspended in 12 mL of acetic acid-sodium acetate buffer (pH 5.0), incubated at 30 ℃, shaken at 200 rpm for 2 h, and then centrifuged at 4 ℃ and 6000 rpm for 5 min (Lu et al. 2023). The supernatant was stored at − 20 °C for enzyme assays. Lignocellulolytic enzyme activities were determined with a BOXBIO kit (Beijing, China). Filter paper enzyme (FPase), endoglucanase, and xylanase activities were assayed by determining the release of reducing sugars generated from the hydrolysis of filter paper, sodium carboxymethylcellulose, and xylan, respectively. β-Glucosidase, exoglucanase, and β-xylosidase activities were detected by measuring the amount of p-nitrophenol generated from p-nitrophenyl-β-D-glucopyranoside, p-nitrophenylcellobioside, and p-nitrophenyl-β-D-xylopyranoside, respectively. Manganese peroxidase (MnP), versatile peroxidase (VP), and laccase (Lac) activities were evaluated by monitoring the oxidation of guaiacol, veratryl alcohol, and 2,2’-azino-bis-3-ethylbenzothiazoline-6-sulfonic acid (ABTS), respectively. One unit (U) of enzyme activity is defined as the quantity of enzyme required to produce 1 μmol of product per minute per kilogram of fermentation sample.

### Scanning electron microscopy (SEM) analysis

Fermented WS samples from the IW and IN groups were collected on Days 0, 5, and 30. To preserve morphological integrity, the samples were immediately fixed in 2.5% glutaraldehyde at 4 °C. Following fixation, the samples were dehydrated through a graded ethanol series and subjected to critical point drying. The dried samples were then sputter-coated with gold and examined under a scanning electron microscope (Guoyi Quantum SEM3200, China).

### Fourier transform infrared spectroscopy (FTIR) analysis

Dried WS samples from different fermentation time points were analyzed using a Nicolet 470 FTIR spectrometer (USA). Absorption spectra were recorded over 400–4000 cm⁻^1^ at a scan rate of 8 cm/s, and each sample was scanned 64 times to improve the signal-to-noise ratio. Baseline correction was applied to the collected spectra to minimize light scattering and other sources of interference, and the corrected spectra were then visualized for analysis.

### X-ray diffraction (XRD) analysis

The crystallinity index (CrI) of WS was determined using a Bruker D8-ADVANCE X-ray diffractometer (Germany). Nickel-filtered Cu-Kα radiation (wavelength of 1.5418 Å) was used, with scanning under 40 kV and 40 mA via θ/2θ mode at 2°/min over 5–90° (Lu et al. 2023). Relative crystallinity was calculated using the Segal method, which is based on diffraction peak intensity (Segal et al. 1959) and defined as follows:

$$ {\mathrm{CrI}}\left( \% \right) = I_{002} {-}I_{am} /I_{am} *{1}00\% $$

where *I*_*002*_ represents the diffraction intensity of the crystalline region (corresponding to 2θ = 22°), and *I*_*am*_ denotes the diffraction intensity of the amorphous region (corresponding to 2θ = 18°).

### Transcriptomic analysis

Mycelia of *I. lacteus* QJ grown on PDA plates at 30 °C for 5 d served as the control (CK). Mycelia from the IW and IN groups were harvested on Days 5, 10, 20, and 30, with three biological replicates for each time point. The samples were promptly frozen in liquid nitrogen and subsequently stored at − 80 °C. Total RNA was extracted using a QIAzol Lysis Reagent kit (Qiagen, Germany), and the concentration and purity of the RNA were assessed using a NanoDrop 2000 spectrophotometer (Thermo Fisher Scientific), with a threshold of A260/A280 > 1.8. The integrity of the RNA was confirmed through 1% agarose gel electrophoresis. A cDNA library was constructed with an Illumina® Stranded mRNA Prep Ligation kit (Illumina, San Diego, CA) and sequenced on the Illumina NovaSeq 6000 platform (Shanghai Majorbio Bio-pharm Technology Co., Ltd). Differentially expressed genes (DEGs) were identified using DESeq2 software with thresholds of padj < 0.05 and |log2(fold change)|≥ 1. DEGs underwent comprehensive functional annotation: protein function annotation was conducted using the Swiss-Prot database; functional classification was based on clusters of orthologous groups (COG); and gene ontology (GO) functional enrichment and Kyoto Encyclopedia of Genes and Genomes (KEGG) pathway enrichment analyses were performed utilizing hypergeometric distribution tests. Finally, multivariate statistical analysis and visualization were conducted on the Majorbio Cloud platform (https://cloud.majorbio.com) (Ren et al. 2022). To further explore the interaction patterns of the DEGs, a protein‒protein interaction (PPI) network was constructed using the STRING database (https://cn.string-db.org) at a composite score threshold ≥ 0.4 and visualized in Cytoscape 3.8.2 (Szklarczyk et al. 2015). Key proteins were identified using CytoHubba on the basis of degree centrality. Proteins with higher degree centrality values have more interactions and greater topological significance in the network (Chin et al. 2014; Shannon et al. 2003). Transcriptomic data have been deposited in the NCBI SRA under BioProject accession number PRJNA1328065.

### Metabolomic analysis

Fermented samples of *I. lacteus* QJ and WS from the IW and IN groups were collected on Days 0, 5, 10, 20, and 30, freeze-dried, and ground into powder. Fifty milligrams of powder was accurately weighed and placed into a 2 mL centrifuge tube, with 6 replicates for each time point. Afterward, 6 mm grinding beads and 400 μL extraction solution (methanol–water at a 4:1 volume ratio, containing the internal standard L-2-chlorophenylalanine at 0.02 mg/mL) were added. The samples underwent cryogenic grinding, followed by low-temperature ultrasonic extraction. After centrifugation, the supernatant was collected for subsequent use. Quality control (QC) samples were prepared by mixing equal volumes of all the supernatants, with one QC sample per 10 samples during LC‒MS/MS analysis to evaluate system stability. Detection was performed on a Thermo Scientific UHPLC-Q Exactive HF-X system with an Agilent SB-C18 column (2.1 × 100 mm, 1.8 μm). The mobile phase consisted of 0.1% (v/v) formic acid in water (Phase A) and 0.1% (v/v) formic acid in acetonitrile (Phase B), with gradient elution (Mao et al. 2023; Zhang et al. 2023a). The raw data were processed for peak extraction, identification, and normalization using Progenesis QI software (Waters Corporation, USA). Multivariate statistical analysis and visualization were performed via the Majorbio Cloud platform (https://cloud.majorbio.com). The metabolomic data have been deposited in the MetaboLights database under the accession number MTBLS13099.

### Real-time quantitative PCR (RT‒qPCR) assays

Seven DEGs related to lignocellulose degradation were selected for RT*‒*qPCR validation, with DN1648_c0_g1 (gamma tubulin) as the reference gene (Supplementary Table S1). Total RNA was reverse-transcribed using the SweScript All-in-One RT SuperMix for qPCR kit (Vazyme, China). RT*‒*qPCR was performed using the SuperReal PreMix Plus kit (TIANGEN, China). Relative expression levels were calculated using the 2^−ΔΔCt^ method (Livak and Schmittgen 2001).

### Data statistics and analysis

All experiments were executed in triplicate, and the mean values were computed. Statistical analyses were carried out using SAS version 9.4. First, the data were tested for normality and homogeneity of variance and then analyzed using a one-way ANOVA. The LSD post hoc test was subsequently used to assess differences among groups; significant differences (*P* < 0.05) are indicated by different letters.

## Results and discussion

### Fungal fermentation-mediated degradation of lignocellulose and enhancement of nutritional value in WS

**Fig. 1:**
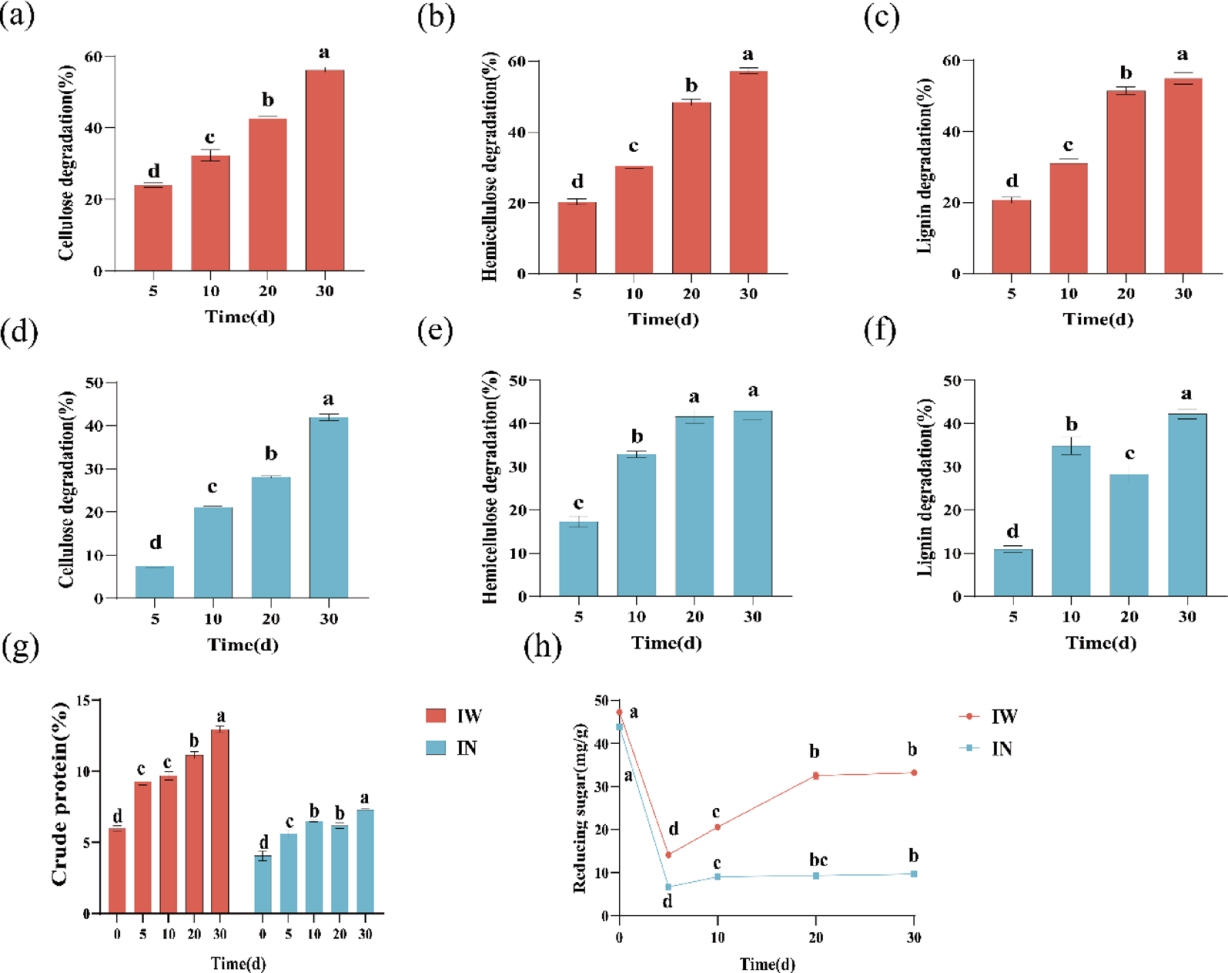
Changes in the lignocellulose degradation rate and nutrient components of WS during SSF with *I. lacteus* QJ. **a**–**c** Degradation rates of cellulose, hemicellulose, and lignin in the IW group, respectively. **d**–**f** Degradation rates of cellulose, hemicellulose, and lignin in the IN group, respectively. **g** Crude protein content in the IW and IN groups. **h** Reducing sugar content in the IW and IN groups. Different letters indicate significant differences among treatments (*P* < 0.05)

The chemical composition of WS was analyzed to evaluate the lignocellulose degradation efficacy of *I. lacteus* QJ. The contents of cellulose, hemicellulose and lignin were significantly reduced by *I. lacteus* QJ in both the IW and IN groups. The NDF, ADF, and CF contents were also markedly decreased, enhancing the palatability of fermented WS as animal feed (Kumar et al. 2008) (Table 1). During the 0–30-day fermentation period, *I. lacteus* QJ gradually reduced the cellulose, hemicellulose, and lignin contents. In the IW group, their contents decreased from 37.95%, 28.41%, and 6.89% to 26.37%, 19.25%, and 4.93%, respectively. In the IN group, the cellulose content decreased from 63.78% to 50.67%, the hemicellulose content decreased from 15.78% to 12.30%, and the lignin content decreased from 7.14 to 5.64%. The degradation rates reached 56.21%, 57.30%, and 54.90% in the IW group and 41.90%, 42.94%, and 42.23% in the IN group (Fig. 1a–f). Furthermore, dry matter loss progressively increased, reaching 36.97% in the IW group and 26.87% in the IN group by Day 30 (Supplementary Fig. S1). In summary, compared with many other reported fungi *I. lacteus* QJ significantly reduced the lignocellulose content in both groups and efficiently and synchronously degraded the three major components. For instance, Ying et al. (2024) reported only 11.8%, 5.9%, and 24.3% degradation of hemicellulose, cellulose, and lignin, respectively, after 15 days using the white-rot fungus *Cerrena unicolor*. Niu et al. (2018) reported 26.01%, 6.16%, and 27.30% degradation of these components by *P. ostreatus* at 28 days. Zeng et al. (2014) found that the rate of lignin degradation by *P. chrysosporium* could exceed 50%; however, the treatment duration exceeded 8 weeks. Similarly, *P. ostreatus* and *Trametes versicolor* achieved 37.48% and 31.29% lignin degradation, respectively, over 30 days (Shrivastava et al. 2011).

**Table 1:** Changes in lignocellulose content during SSF by *I. lacteus* QJ

Group	Time (d)	NDF (%)	ADF (%)	CF (%)	Cellulose (%)	Hemicellulose (%)	Lignin (%)
IW	0	73.26 ± 0.20^a^	44.85 ± 0.75^a^	39.32 ± 0.14^a^	37.95 ± 0.64^a^	28.41 ± 0.56^a^	6.89 ± 0.16^a^
	5	65.26 ± 0.17^b^	39.32 ± 0.28^b^	33.96 ± 0.59^b^	33.05 ± 0.29^b^	25.94 ± 0.28^b^	6.27 ± 0.07^b^
	10	62.78 ± 0.77^c^	38.08 ± 0.68^c^	32.00 ± 0.73^c^	32.13 ± 0.75^bc^	24.70 ± 0.26^c^	5.95 ± 0.11^c^
	20	57.47 ± 0.60^d^	36.31 ± 0.46^d^	26.86 ± 0.22^d^	31.49 ± 0.41^c^	21.16 ± 0.36^d^	4.82 ± 0.11^d^
	30	50.56 ± 0.45^e^	31.30 ± 0.46^e^	23.68 ± 0.78^e^	26.37 ± 0.40^d^	19.25 ± 0.38^e^	4.93 ± 0.17^d^
IN	0	86.69 ± 0.20^a^	70.92 ± 0.29^a^	64.87 ± 0.04^a^	63.78 ± 0.15^a^	15.78 ± 0.33^a^	7.14 ± 0.15^a^
	5	81.24 ± 0.49^b^	67.71 ± 0.29^b^	61.62 ± 1.35^b^	61.12 ± 0.34^b^	13.52 ± 0.21^b^	6.60 ± 0.06^b^
	10	78.78 ± 0.48^c^	66.06 ± 0.35^c^	60.03 ± 0.90^b^	60.47 ± 0.21^c^	12.72 ± 0.14^c^	5.59 ± 0.17^c^
	20	76.26 ± 0.06^d^	64.60 ± 0.33^d^	57.67 ± 1.52^c^	58.11 ± 0.20^d^	11.66 ± 0.32^d^	6.49 ± 0.18^b^
	30	68.62 ± 0.41^e^	56.31 ± 0.70^e^	49.95 ± 0.29^d^	50.67 ± 0.64^e^	12.30 ± 0.43^c^	5.64 ± 0.11^c^

The data are presented as the mean ± standard deviation of three replicates. The different letters indicate significant differences (*P* < 0.05)

Crude protein serves as both a key nutrient in feed and an indicator of the lignocellulose degradation efficacy of *I. lacteus* QJ. In both groups, the crude protein content increased significantly with increasing fermentation time. In the IW group, the crude protein content was 5.98% on Day 0 and increased significantly to 9.30% on Day 5. There was no significant difference from Day 5 to Day 10. The content then increased further to 11.13% on Day 20 and peaked at 12.96% on Day 30, representing a total increase of 116.7%. In the IN group, the crude protein content was 4.05% on Day 0. It increased significantly to 5.61% on Day 5 and increased further to 6.50% on Day 10. The crude protein content peaked at 7.07% on Day 30, which was a 74.6% greater than the initial level (Fig. 1g). The increase in the crude protein content of WS mediated by *I. lacteus* QJ exceeded that previously reported for *P. ostreatus* and *T. versicolor* (Shrivastava et al. 2011). *I. lacteus* QJ degraded lignocellulose for its growth and metabolism, which led to a decrease in the dry matter content of WS, increased the mycelial protein content, and ultimately increased the crude protein content of the fermentation system.

The reducing sugar concentration sharply decreased during the initial phase (0–5 d) in both groups. In the IW group, the reducing sugar content increased significantly from Days 5–20 but then increased slowly from Days 20–30. In the IN group, the reducing sugar content increased significantly from Days 5–10 but then increased slowly from Days 10–20 and 20–30 (Fig. 1h). These findings indicate that *I. lacteus* QJ initially uses reducing sugars in the fermentation nutrient solution as the primary carbon source for growth and reproduction, which decreased the reducing sugar concentration during the early stage of SSF. As fermentation progressed, the percentage of reducing sugars released from *I. lacteus* QJ-mediated lignocellulose degradation exceeded that consumed by fungal metabolism, ultimately increasing the concentration to 33.18 mg/g in the IW group and 9.63 mg/g in the IN group. This dynamic profile suggests that sugar production rates persistently surpass metabolic consumption and that the level of sugar accumulation is positively correlated with lignocellulose degradation efficiency. These findings are consistent with the work of Dorado et al. (1999), who reported that the reducing sugar content in fermentation substrates stabilized eventually during the 0–60 d SSF of WS with *Pleurotus eryngii* and *Phlebia radiata*.

Interestingly, all the measured indicators, including the degradation rates of cellulose, hemicellulose, and lignin; dry matter loss; crude protein content; and reducing sugar concentration, were higher in the IW group than in the IN group (Fig. 1). A possible explanation is that NaOH pretreatment significantly increased the cellulose content and reduced the hemicellulose content in WS (Supplementary Fig. S2), a phenomenon that is consistent with reports by Jeya et al. (2009), Singh et al. (2019) and Rahnama et al. (2014). Such compositional changes may affect the lignocellulose degradation efficiency of fungi (Monlau et al. 2012; Rouches et al. 2016), resulting in a lower accumulation of crude protein and reducing sugars in the IN group than in the IW group. In summary, *I. lacteus* QJ exhibits excellent lignocellulose-degrading ability and can simultaneously degrade multiple lignocellulosic components. It serves as a promising candidate strain for straw feed preparation and biomass substrate conversion. This strain is well suited for degrading natural lignocellulose, thereby effectively avoiding the higher costs.

### Changes in enzyme activity

**Fig. 2:**
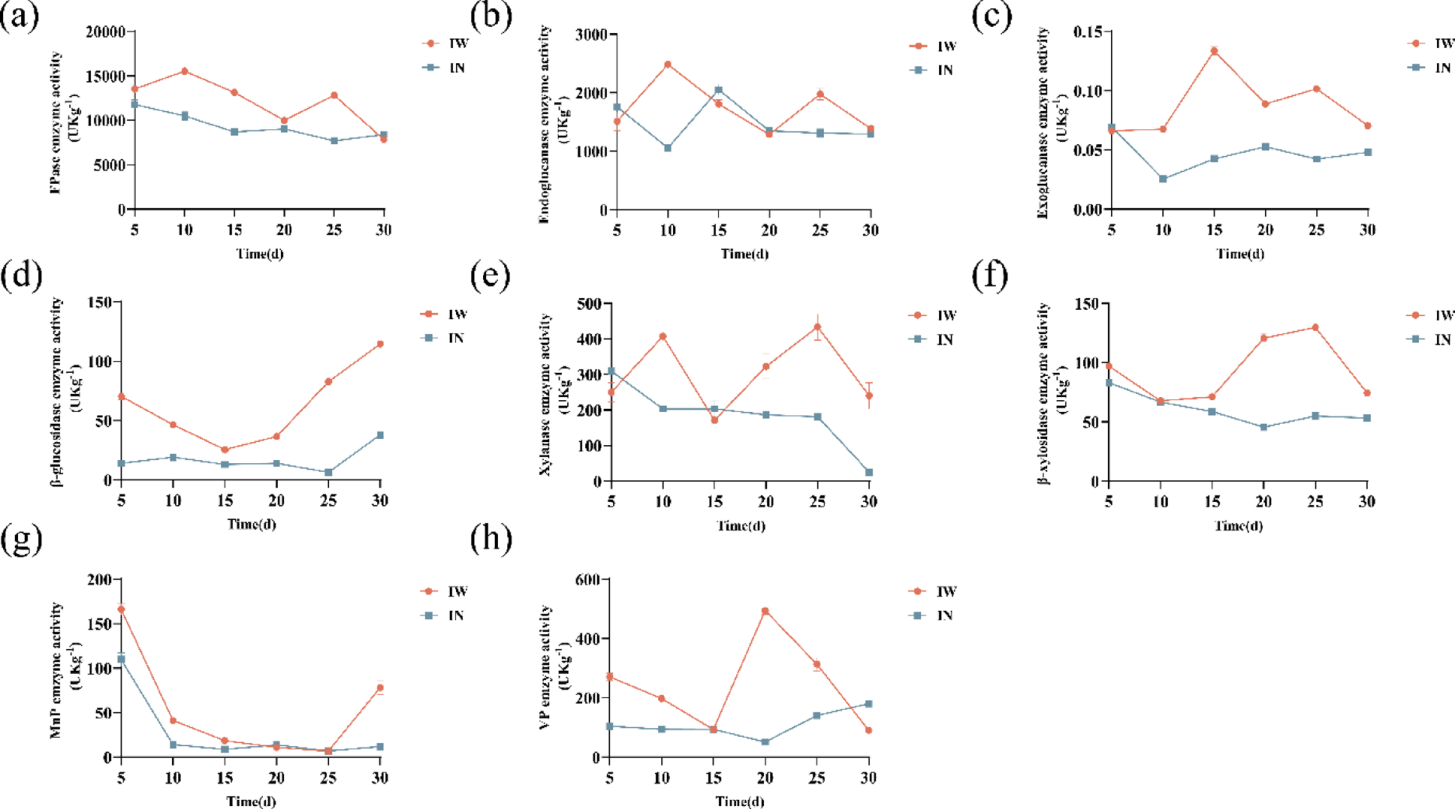
Enzyme activities of the lignocellulolytic enzymes produced by *I. lacteus* QJ in SSF. **a** FPase, **b** Endoglucanase, **c** Exoglucanase, **d** β-glucosidase, **e** Xylanase, **f** β-xylosidase, **g** MnP, and **h** VP

To understand the lignocellulose degradation mechanism of *I. lacteus* QJ, dynamic changes in extracellular lignocellulolytic enzymatic activities were monitored during WS decomposition. The activities of FPase, endoglucanase, exoglucanase, and β-glucosidase were determined for cellulose degradation. FPase activity represented total cellulase activity and the other three were the main cellulases. In the IW group, FPase and endoglucanase activities peaked on Day 10 (15,525.13 U/kg and 2483.75 U/kg, respectively; Fig. 2a and b), exoglucanase activity peaked 0.13 U/kg on Day 15 (Fig. 2c), and β-glucosidase activity peaked at 114.60 U/kg by Day 30 (Fig. 2d). In contrast, the IN group exhibited earlier peaks for FPase (11,781.71 U/kg on Day 5; Fig. 2a) and exoglucanase (0.07 U/kg on Day 5; Fig. 2b), whereas endoglucanase and β-glucosidase peaked later (2053.87 U/kg on Day 15 and 37.96 U/kg on Day 30, respectively; Fig. 2c and d). Notably, the IW group exhibited higher cellulase activities, which is consistent with its superior cellulose degradation rates. Compared with the maximum FPase activity of 5.83 U/g reported for *Aspergillus niger* during the SSF of WS at the optimal moisture content (Pensupa et al. 2013) and 0.67 U/g for *Aspergillus tubingensis* JP-1 (Pandya and Gupte 2012), *I. lacteus* QJ not only exhibits superior cellulase activity but also has a greater variety of cellulases.

With respect to hemicellulose degradation, xylanase and β-xylosidase, which are key enzymes responsible for hydrolyzing hemicellulose into xylose, exhibited distinct temporal profiles in the two groups. In the IW group, xylanase activity increased during the first 10 days, decreased transiently, and then increased again to a maximum of 433.52 U/kg on Day 25 (Fig. 2e). Conversely, xylanase activity in the IN group gradually decreased after Day 5, peaking at 309.17 U/kg (Fig. 2e). The β-xylosidase activity in the IW group initially decreased but then progressively increased to 129.64 U/kg by Day 25, whereas that in the IN group reached its maximum (83.02 U/kg) on Day 5 (Fig. 2f). Like cellulase activity, the IW group exhibited higher hemicellulase activity than the IN group, which is consistent with its superior hemicellulose degradation rate. Most white-rot fungi are not renowned for their hemicellulase activity, but *I. lacteus* QJ exhibited significantly higher xylanase activity than the white-rot fungus *Pleurotus pulmonarius* when the latter was cultivated on a mixed substrate of corn cob, soybean meal, and wheat bran (Corrêa et al. 2016).

For lignin degradation, MnP, VP and Lac were assayed. MnP activity peaked on Day 5 in both the IW (166.17 U/kg) and IN groups (109.81 U/kg; Fig. 2g), indicating rapid peroxidase activation in the early stage (Gupta et al. 2023; Huang et al. 2020). VP activity in the IW and IN groups peaked on Days 20 (494.63 U/kg) and 30 (180.08 U/kg), respectively (Fig. 2h). The higher ligninolytic enzyme activity in the IW group corresponds with its higher lignin degradation rate. However, no Lac activity was detected in either group, which is consistent with previous reports that the *Lac* gene is absent in the *I. lacteus* CD2 genome (Qin et al. 2018).

Overall, *I. lacteus* QJ can secrete a comprehensive suite of lignocellulolytic enzymes and is an excellent lignocellulose-degrading fungus. Additionally, the activities of cellulase, hemicellulase, and ligninase were consistently higher in the IW group than in the IN group throughout SSF, aligning with the greater superior degradation rates of cellulose, hemicellulose, and lignin observed in the IW group. The observed differences in enzyme activity and degradation efficiency between the two groups may be attributed to the fact that although NaOH pretreatment effectively removes lignin, it may also adversely affect the physical structure of cellulose. This inference was supported by subsequent XRD analysis, which revealed a significant increase in cellulose CrI in the IN group, thereby forming a physical barrier that hinders access by cellulolytic enzymes (Bertran and Dale 1985; Hall et al. 2010). The results suggest that *I. lacteus* QJ can secrete a wide range of lignocellulolytic enzymes with excellent activities, demonstrating its potential application in the development of lignocellulolytic enzymes.

### SEM analysis

**Fig. 3:**
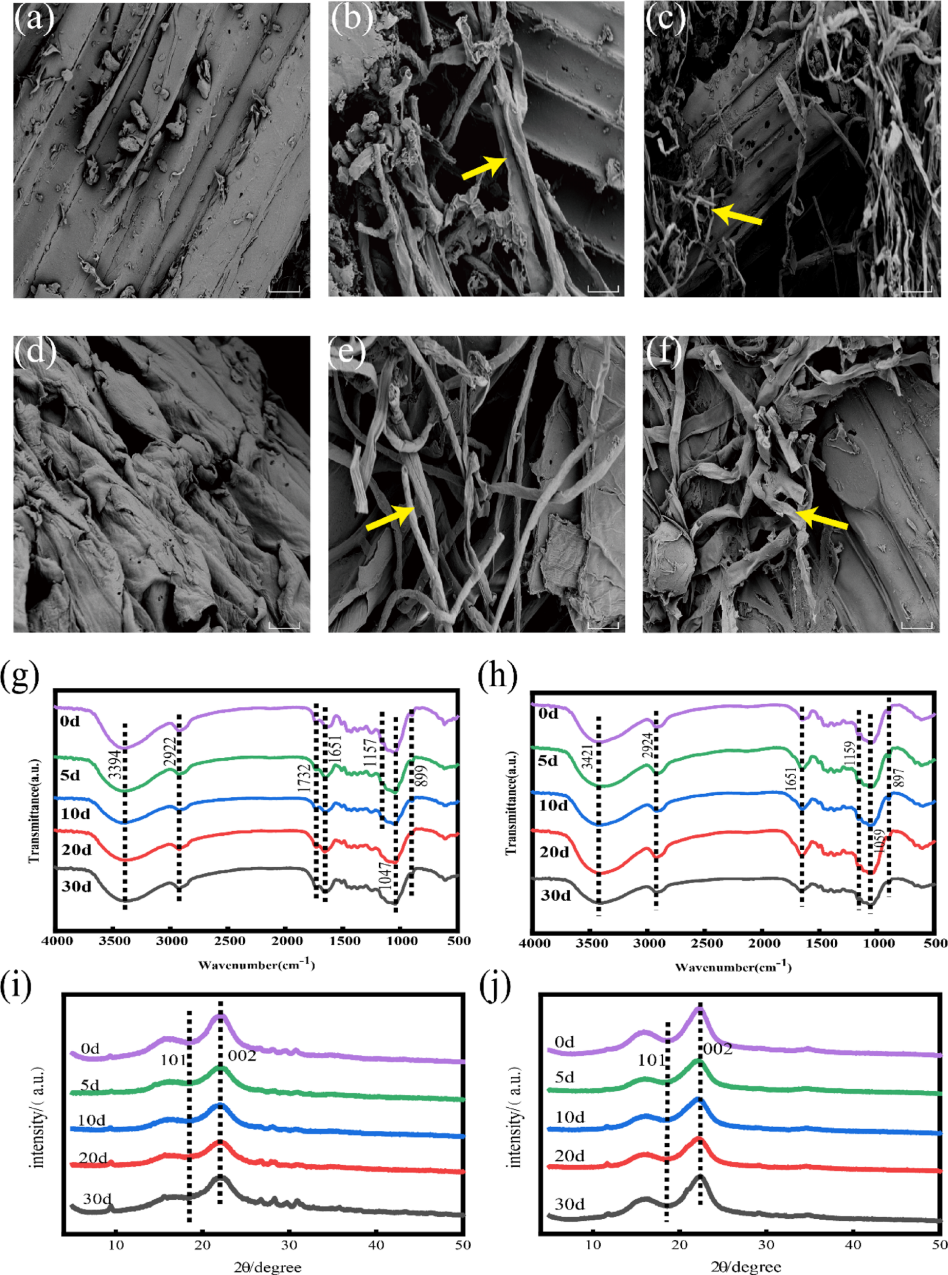
Changes in the structural characterization of WS during SSF by *I. lacteus* QJ. **a**–**c** SEM images of the IW group at Days 0 **a**, 5 **b** and 30 **c**, respectively. **d**–**f** SEM images of the IN group at Days 0 (**d**), 5 (**e**) and 30 (**f**), respectively. The bars in the SEM images represent 10 μm. FTIR spectra of WS in the IW group (**g**) and the IN group (**h**). X-ray diffraction patterns for the cellulose crystallinity of WS in the IW group (**i**) and IN group (**j**). The yellow arrow indicates hyphae that are degrading and entwining the WS

SEM revealed similar alterations in WS surface morphology and hyphae–WS interactions in both groups during fermentation by *I. lacteus* QJ. The surface of raw WS (Day 0) was flat and intact (Fig. 3a), whereas that of NaOH-pretreated WS (Day 0) became wrinkled and exhibited more micropores (Fig. 3d). By Day 5, *I. lacteus* QJ had developed robust hyphae that mechanically disrupted the WS surface, with hyphae invading and enlarging the pores, further penetrating into the internal cavities in both groups (Fig. 3b and e). By Day 30, the hyphae had extensively colonized both the surface and interior of the WS, resulting in structural loosening and localized fragmentation in both groups (Fig. 3c and f). These observations are consistent with previous reports that microorganisms adhere to and grow on biomass surfaces while penetrating pores and interstices (Zhang et al. 2023b). The results indicate that *I. lacteus* QJ breaks down WS by synergistic action, in which the hyphae physically disrupt WS and simultaneously secrete enzymes to chemically degrade the lignocellulose.

### FTIR analysis

The characteristic FTIR spectral peaks of WS are shown in Fig. 3g and h. A broad absorption band at 3300–3500 cm^−1^ corresponds to O–H stretching vibrations in cellulose, hemicellulose, and polysaccharides. The peak at 2900–2935 cm⁻^1^ is attributed to asymmetric C–H stretching of the CH_3_ and CH_2_ groups in cellulose (Xu et al. 2013). Absorption at 1640–1735 cm^−1^ arises from C=O stretching in carboxylic esters and ketones of lignin or hemicellulose (Dayo et al. 2018). The peak at 1035–1200 cm^−1^ is related to C–O–C asymmetric stretching in cellulose and hemicellulose (Kumar et al. 2009), whereas the peak at 970–1100 cm^−1^ corresponds to C-O stretching in polysaccharides (Kumar et al. 2009). The peak at 890–900 cm^−1^ is characteristic of β-D-glucosidic linkages and intramolecular hydrogen bonds (Zhao et al. 2013). Compared with raw WS (0 d), NaOH-pretreated WS (0 d) had no covalent bond intensities at 1105 cm^−1^ and 1732 cm^−1^ (Supplementary Fig. S3). Since these peaks are associated with hemicellulose, their disappearance is consistent with the reduction in hemicellulose content after NaOH pretreatment (Supplementary Fig. S2). These findings align with the report by Rahnama et al. (2014) that alkaline pretreatment facilitates the removal of hemicellulose. During SSF, changes in relative transmittance intensity indicated dynamic lignocellulose degradation (Supplementary Tables S2 and S3). The peak at 1157 cm^−1^ disappeared in the late stage of fermentation in the IW group, indicating complete cleavage of the C–O–C bond in cellulose and hemicellulose by *I. lacteus* QJ.

### XRD analysis

The XRD patterns of WS in the IW and IN groups during fermentation are shown in Fig. 3i and j. The crystallinity of raw WS was 48.61%, which increased to 58.24% after NaOH pretreatment. This result agrees with the reports by Liu et al. (2009) and Barl et al. (1986) that acid or alkaline pretreatment of corn stover led to increased crystallinity. This is because NaOH pretreatment removes the amorphous components of lignocellulose, thereby increasing the CrI of NaOH-pretreated WS (Chen et al. 2023; Wu et al. 2022). By Day 30, the crystallinity of WS was 44.33% and 56.29% in the IW group and IN group, respectively. The results revealed that the crystallinity of WS decreased in both groups after fermentation by *I. lacteus* QJ, and this change was correlated with a reduction in the cellulose content. However, the magnitude of the decrease in crystallinity was smaller in the IN group than in the IW group. This difference indicated that the higher initial crystallinity reduced the degradation efficiency of *I. lacteus* QJ on WS. Cellulose crystallinity is a key parameter that determines its biodegradability. In crystalline regions, the molecular chains are tightly packed with a stable hydrogen-bonding network, rendering cellulases largely inaccessible; thus, degradation preferentially occurs in the more disordered amorphous regions. Consequently, higher initial crystallinity results in lower enzymatic accessibility, leading to lower degradation efficiency (Hall et al. 2010). This principle has been widely validated across various biodegradation systems. Rudakiya and Gupte (2019) found that compared with high-crystallinity residues obtained from *Tricholoma giganteum* AGDR1, hardwood cellulose treated with *Pseudolagarobasidium acaciicola* AGST3 exhibits a fourfold reduction in crystallinity, resulting in a significantly greater degradation efficiency. Moreover, in enzymatic hydrolysis, chemical hydrolysis, and heterogeneous catalytic degradation of polymeric materials, high crystallinity similarly inhibits the degradation efficiency by decreasing substrate accessibility and reducing the number of available reaction sites (Dhayer et al. 2025; Jang et al. 2024; Wang et al. 2022).

### Transcriptomic analysis

#### Transcriptome sequencing and differential expression analysis

To further investigate the lignocellulose degradation mechanism, transcriptome sequencing analysis was performed on *I. lacteus* QJ grown on raw WS and NaOH-pretreated WS at different time points. A total of 235.64 Gb of filtered bases were obtained from 27 samples. The Q20 and Q30 values were greater than 98% and 95%, respectively, indicating high sequencing quality that is suitable for subsequent analyses (Supplementary Table S4). In total, 9921 unigenes and 45,744 transcripts were identified, representing a comprehensive repertoire of transcriptional regulatory components for this strain in response to the substrates (Supplementary Table S5). Principal component analysis (PCA) confirmed good reproducibility among the biological replicates (Supplementary Fig. S4a and d). In the IW and IN groups, the samples were clearly separated according to SSF time points, indicating that the degradation stage induced substantial transcriptional reprogramming. By comparing DEGs across time points between the two groups, key genes associated with lignocellulose degradation can be identified. Compared with CK, the IW group had 488, 785, 802, and 1710 upregulated genes and 580, 285, 451, and 1550 downregulated genes on Days 5, 10, 20, and 30, respectively (Supplementary Fig. S4b). Similarly, the IN group had 552, 839, 931, and 1022 upregulated genes and 285, 573, 759, and 638 downregulated genes on the corresponding days (Supplementary Fig. S4e). Venn analysis further revealed 190 common DEGs (109 upregulated, 81 downregulated) and 379 (278 upregulated, 101 downregulated) across different time points within each group (Supplementary Fig. S4c and f).

#### DEGs analysis and identification of core protein-coding genes

**Fig. 4:**
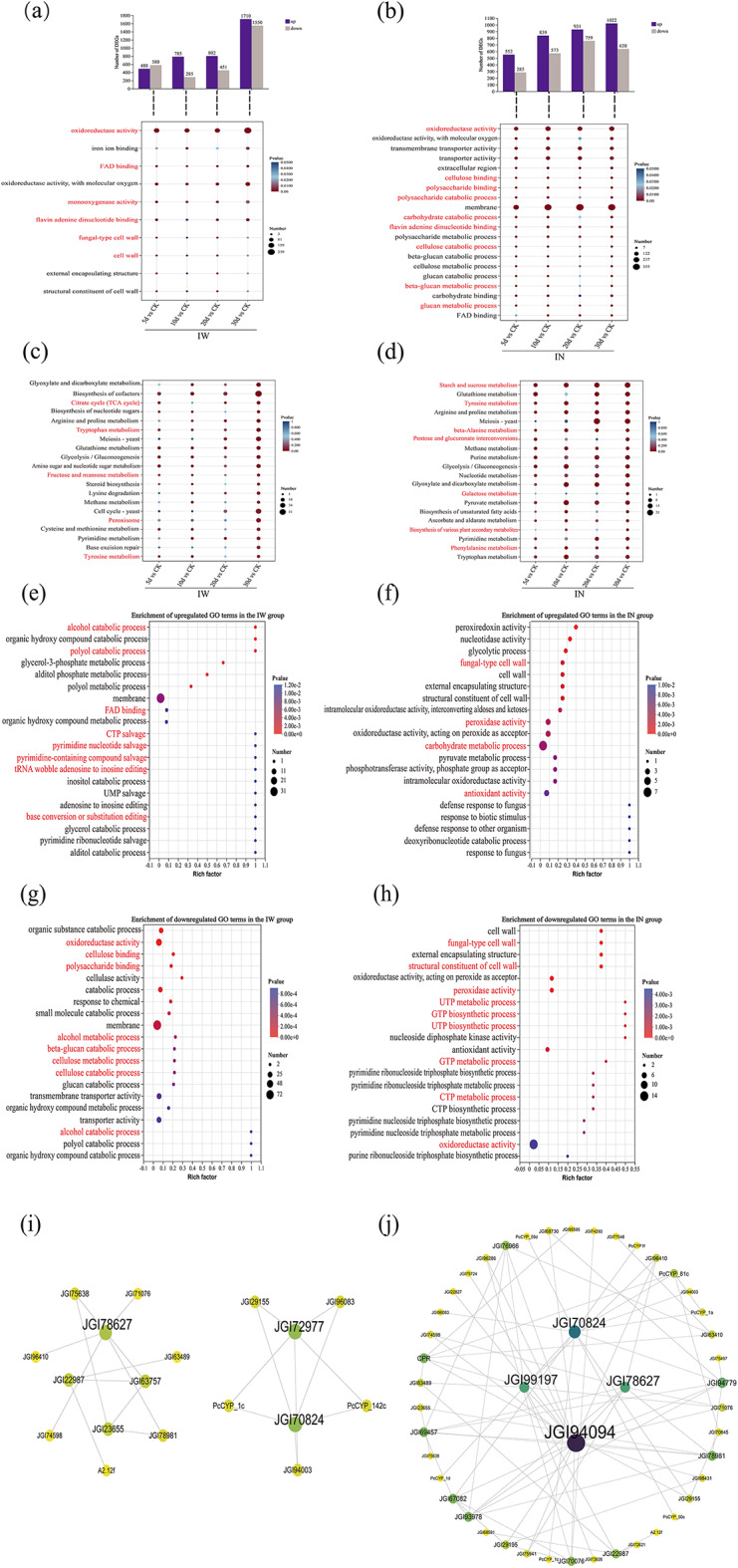
GO and KEGG analyses of DEGs and PPI network analysis. **a**, **b** Shared GO terms enriched by DEGs at each fermentation stage in the IW (**a**) and IN (**b**) groups (*P* < 0.05). **c**, **d** Shared KEGG pathways enriched by DEGs at each fermentation stage in the IW (**c**) and IN (**d**) groups. The top 20 enriched KEGG pathways are shown in the figure, in which a redder color indicates a lower *P* value and more significant enrichment. **e**, **f** GO terms enriched by the commonly upregulated (**e**) and downregulated (**f**) DEGs in the IW group (*P* < 0.05). **g**, **h** GO terms enriched by the common upregulated (**g**) and downregulated (**h**) DEGs in the IN group (*P* < 0.05). **i**, **j** Core protein network analysis of the common upregulated DEGs in the IW (**i**) and IN (**j**) groups. Each node represents a protein annotated in the STRING database. The darkness of the color and size of the node are proportional to the number of interactions. A darker color and larger size indicate a higher degree centrality for the node. The red text indicates the key GO terms and metabolic pathways discussed in this study

GO enrichment analysis revealed that the DEGs in the IW group were mainly involved in terms such as oxidoreductase activity, flavin adenine dinucleotide binding, monooxygenase activity, and cell wall interactions (Fig. 4a). Conversely, GO terms for the IN group significantly enriched in biological processes included oxidoreductase activity, cellulose binding, polysaccharide binding, polysaccharide catabolic processes, carbohydrate catabolic processes, flavin adenine dinucleotide binding, β-glucan catabolic processes, cellulose catabolic processes, and glucan metabolic processes (Fig. 4b). These enriched processes in both groups are related to lignocellulose degradation (Huang et al. 2022).

KEGG pathway analysis revealed that *I. lacteus* QJ adopted distinct metabolic strategies for degradation in the IW and IN groups. In the IW group, the DEGs were mainly enriched in the TCA cycle, amino acid metabolism, fructose and mannose metabolism, and the peroxisome pathway (Fig. 4c). The TCA cycle provides ATP and NADH for cellulases, hemicellulases, and peroxidases (VP, MnP), thereby facilitating cellulose/hemicellulose hydrolysis and lignin oxidative depolymerization. Its intermediates also enter amino acid synthesis, promoting mycelial growth. The peroxisome pathway is enriched in H_2_O_2_‑producing enzymes, which supply cofactors for MnP and VP and mediate lignin bond cleavage (Kersten and Kirk 1987). In the IN group, the DEGs were mainly enriched in starch and sucrose metabolism, pentose and glucuronate interconversions, galactose metabolism, biosynthesis of various plant secondary metabolites, and amino acid metabolism (Fig. 4d). The pentose and glucuronate interconversion pathway corresponds to the degradation of xylan and galacturonic acid residues, whereas the coordinated activation of starch/sucrose metabolism and galactose metabolism indicates the efficient conversion of cellulose components into fermentable sugars (Song et al. 2025). The amino acid metabolism pathway, which was enriched in both groups, was consistent with the increase in crude protein content in the fermented WS (Fig. 1g). The activation of these polysaccharide/monosaccharide catabolic pathways in both groups accounts for the rapid conversion of lignocellulose hydrolysis products and the gradual accumulation of reducing sugars (Fig. 1h). In summary, although the enriched KEGG pathways differed markedly between the two groups, these results highlight the coordinated expression of degradation‑related functional genes in *I. lacteus* QJ, which drives the efficient and simultaneous breakdown of cellulose, hemicellulose, and lignin.

The 109 common upregulated DEGs in the IW group were mainly enriched in pathways such as the pyrimidine nucleotide salvage pathway, RNA editing and modification, glycerol and polyol catabolism, and FAD binding (Fig. 4e). Among these pathways, the pyrimidine nucleotide salvage pathway fulfills the basic growth requirements of fungi during WS degradation in an energy-efficient manner (Bernhard et al. 2023); RNA editing and modification ensure the synthesis efficiency of proteins necessary for growth and degradation-related enzymes (Nombela et al. 2021); glycerol and polyol catabolism provides energy for lignocellulolytic enzyme synthesis (Shi et al. 2018); and the upregulation of FAD binding directly correlates with the enhanced activity of FAD-dependent oxidoreductases, including lignin-degrading enzymes. The 278 common upregulated DEGs in the IN group were mainly enriched in pathways related to polysaccharide utilization, alcohol and organic hydroxyl compound metabolism, and oxidoreductase activity (Fig. 4g). The activation of these pathways was closely associated with decreases in cellulose and hemicellulose levels (Table 1) as well as changes in lignocellulolytic enzyme activity during degradation (Fig. 2). While common upregulated DEGs in both groups were involved in lignocellulose degradation-related pathways, a key distinction was that the IW group exhibited a stronger emphasis on growth support and energy metabolism, reflecting more vigorous fungal growth and higher energy demands. This finding was consistent with the phenotypic results showing generally superior lignocellulose degradation rates, dry matter loss, and enzyme activities in the IW group compared with those in the IN group (Figs. 1 and 2). Additionally, KEGG pathway analysis indicated that the upregulated DEGs in both groups were enriched in amino acid metabolism (Supplementary Fig. S5), which is consistent with the aforementioned inference that increased amino acid metabolic activity promotes microbial protein synthesis, thereby contributing to crude protein accumulation in WS during SSF by *I. lacteus* QJ (Fig. 1g).

In the IW group, the 81 common downregulated DEGs were primarily related to peroxidase activity, fungal-type cell wall, carbohydrate metabolic processes, and antioxidant activity (Fig. 4f). Similarly, in the IN group, the 101 common downregulated DEGs were involved in fungal-type cell wall synthesis, ribonucleotide metabolism, and antioxidant activity (Fig. 4h). Compared with those in CK group, genes involved in cell wall synthesis were significantly downregulated in both fermented groups. This phenomenon may reflect an adaptive adjustment of growth metabolism in *I. lacteus* QJ under the stress of using lignocellulose as the sole carbon source, potentially enabling the reallocation of more metabolic resources toward the synthesis and secretion of lignocellulolytic enzymes. This observation is consistent with the metabolic regulation of filamentous fungi under carbon-source stress proposed by Kubicek et al. (2014), who suggested that fungi tend to prioritize allocating energy and materials to secreting degradative enzymes for carbon acquisition when growing on complex carbon sources. Furthermore, the reduced antioxidant activity in both groups, including relatively low expression of the peroxiredoxin gene (*Prx1*) (Supplementary Table S6), may facilitate the accumulation of H_2_O_2_, which activates lignin-degrading enzymes and enhances lignin degradation efficiency (Li et al. 2021). These results reveal the molecular mechanism through which *I. lacteus* QJ optimizes the efficiency of lignocellulose degradation through metabolic resource reallocation.

The above analysis indicates that commonly upregulated DEGs are closely associated with lignocellulose degradation. Therefore, PPI networks were constructed for these genes to identify key hub proteins. In the IW group (Fig. 4i; Supplementary Table S7), the core protein network was dominated by JGI70824 (phenol hydroxylase) and JGI72977 (flavin-dependent monooxygenase), which synergistically drive the oxidative degradation of lignin (Adewale et al. 2021). JGI70824 is a key enzyme that initiates phenolic biodegradation, which catalyzes the hydroxylation of phenol to catechol; the latter is then cleaved by dioxygenase (Radziff et al. 2021; Xiao et al. 2015). JGI72977 catalyzes the hydroxylation, epoxidation, and Baeyer–Villiger oxidation of aromatic rings, thereby leading to a decrease in lignin content after fermentation (Vicente et al. 2015). Additionally, several other key proteins were included in the PPI networks. JGI78627 (a probable D-xylulose reductase) is closely related to hemicellulose degradation. JGI23655 (glycerol kinase) and JGI63757 (glycerol-3-phosphate dehydrogenase) increase carbon source utilization efficiency by coupling glycerol metabolism with the TCA cycle, thereby providing ATP and NADH to support the synthesis of lignocellulose-degrading enzymes. JGI22987 (tyrosinase) may participate in the oxidation of phenolic compounds and cleave the 4-O-5 and Cα-Cβ bonds in lignin model compounds (Min et al. 2017). In the IN group, the core protein network was centered on JGI94094 (aldehyde dehydrogenase), JGI99197 (peroxidase-1), JGI70824, and JGI78627 (Fig. 4j; Supplementary Table S8). JGI94094 converts aldehydes such as vanillin and coniferaldehyde that are produced from lignin oxidation into nontoxic carboxylic acids, thereby maintaining degradation efficiency and alleviating cytotoxicity (Min et al. 2017; Tan et al. 2022). Core protein network analysis of both the IW and IN groups revealed that JGI70824 and JGI78627 play significant and conserved roles in lignocellulose degradation by *I. lacteus* QJ.

#### Validation of DEGs expression levels by RT‒qPCR

Based on transcriptomic analysis, seven DEGs involved in lignocellulose degradation were selected for further validation. RT‒qPCR was performed to determine the expression levels of these genes. The results (Supplementary Fig. S6) revealed that the relative expression trends of these DEGs were largely consistent with the transcriptomic sequencing data (Supplementary Table S1), confirming the reliability of the transcriptomic analysis.

### Metabolomic analysis

#### Quality control of the metabolomic data

To elucidate the response mechanism and identify metabolites associated with lignocellulose degradation by *I. lacteus* QJ, metabolomics analysis was conducted. Both the PCA results and partial least squares discriminant analysis (PLS-DA) revealed excellent intragroup clustering and clear intergroup separation for the IW and IN groups across all the fermentation time points (Supplementary Fig. S7a–d), confirming high reproducibility and significant metabolic differences. The time-dependent sample separation indicates gradual lignocellulose degradation and dynamic changes in metabolite profiles within the straw-fungus system during SSF by *I. lacteus* QJ. These findings lay an important metabolic foundation for the subsequent screening of key metabolites closely related to lignocellulose degradation and for elucidating the regulatory mechanisms of core metabolic pathways. All the models were validated with 200 permutation tests, with the Q^2^ regression line intercepts below 0.05, demonstrating model stability, reliability, absence of overfitting, and suitability for screening differentially expressed metabolites (DEMs) (Supplementary Fig. S7e and f).

#### Analysis of differentially expressed metabolites

The number of upregulated DEMs was greater than that of downregulated DEMs in both groups, with this difference being more pronounced in the IN group (Supplementary Fig. S8a and c). Venn analysis identified 1626 common DEMs in the IW group (825 upregulated, 801 downregulated) and 1341 in the IN group (1193 upregulated, 148 downregulated) (Supplementary Fig. S8b and d). A classification analysis of DEMs was subsequently performed using the Human Metabolome Database (HMDB). The results revealed that the main compound classes, each accounting for more than 5% of the total DEMs included lipids and lipid-like molecules, organic acids and their derivatives, organoheterocyclic compounds, phenylpropanoids and polyketides, organic oxygen compounds, and benzenoids. Throughout SSF (Days 5–30), these six compound classes consistently accounted for more than 5% of the DEMs in both the IW and IN groups, with the classes of lipids and lipid-like molecules remaining predominant, indicating their close association with the lignocellulose degradation process by *I. lacteus* QJ (Supplementary Fig. S9b–i). Notably, lipids and lipid-like molecules constituted more than 20% of the DEMs between the IN and IW groups on Day 0 (Supplementary Fig. S9a). These metabolic findings align with the results of the transcriptome analyses in this study, which revealed that the DEGs commonly upregulated by *I. lacteus* QJ during lignocellulose degradation were enriched in genes related to glycerol and polyol catabolism, potentially contributing to the increased diversity of lipid and lipid-like molecules. Previous studies have demonstrated that *Rhodococcus opacus* can synthesize lipids through aromatic ring cleavage of lignin-derived aromatic compounds (Kosa and Ragauskas 2012; Mahan et al. 2017; Shields-Menard et al. 2018).

**Fig. 5:**
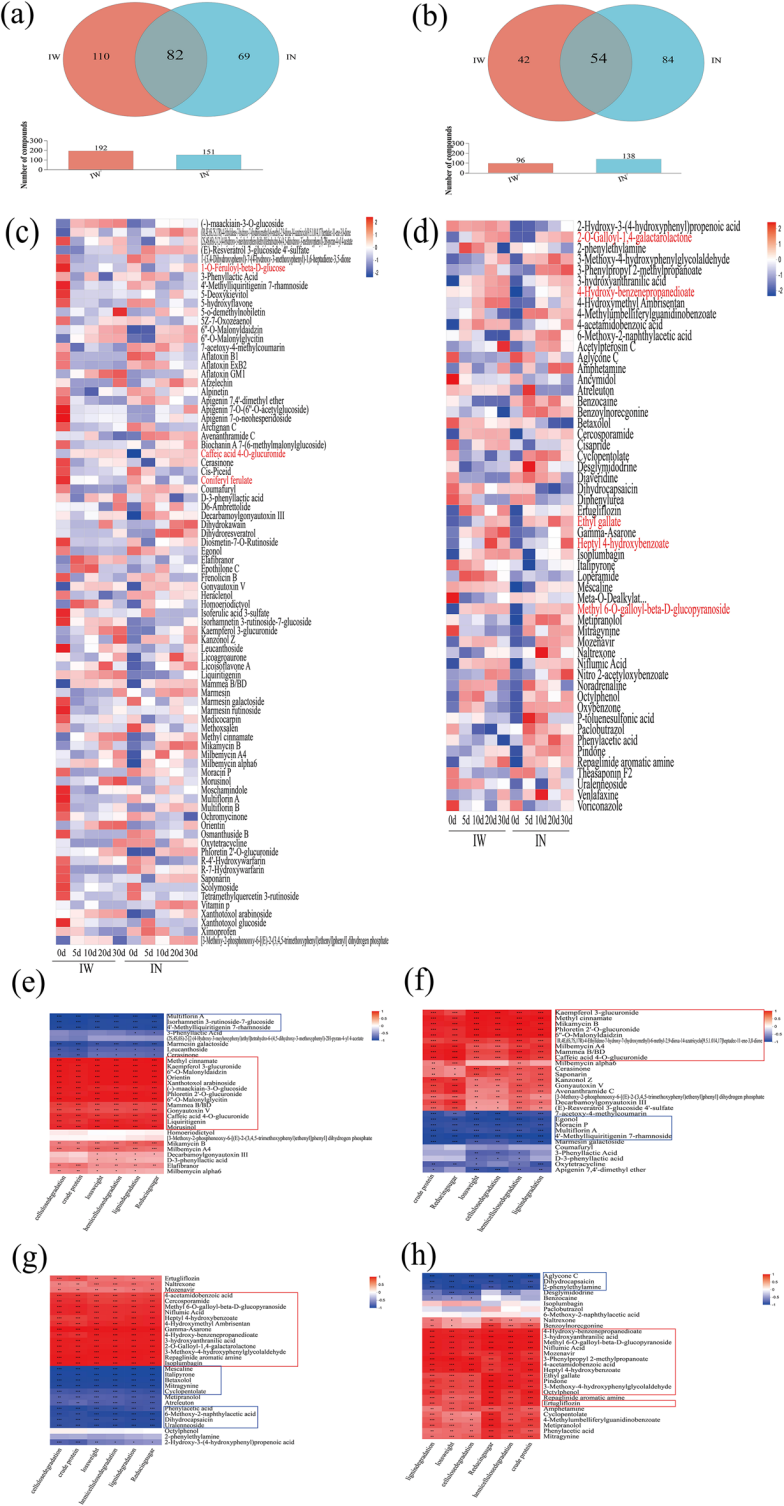
Comparative analysis of DEMs in phenylpropanoids and polyketides versus benzenoids. **a** Venn diagram and dendrogram of DEMs for phenylpropanoids and polyketides in the IW and IN groups. **b** Venn diagram and dendrogram of DEMs for benzenoids in the IW and IN groups. **c**, **d** Heatmap showing the relative expression of common DEMs in phenylpropanoids and polyketides (**c**) and benzenoids (**d**). (**e**, **f**) Correlation heatmaps of common DEMs in phenylpropanoids and polyketides for the IW (**e**) and IN (**f**) groups. **g**, **h** Correlation heatmaps of common DEMs in benzenoids for the IW (**g**) and IN (**h**) groups. Metabolites in the red and blue boxes indicate positive and negative correlations with the phenotype, respectively. The screening criteria for metabolites in the boxes of correlation heatmaps were set as an absolute value of the correlation coefficient > 0.6 and a significance level of* P* < 0.05. The asterisks indicate significance levels. * denotes 0.01 < *P* ≤ 0.05, ** denotes 0.001 < *P* ≤ 0.01, and *** denotes *P* ≤ 0.001

Given that lignin, phenylpropanoids and polyketides, and benzenoids are related to the structural composition of lignin (Wang et al. 2019), the latter two classes of DEMs were selected for further analysis. Among phenylpropanoids and polyketides, 192 DEMs were identified in the IW group and 151 DEMs in the IN group, with 82 DEMs common to both groups (Fig. 5a). The common DEMs included 1-O-feruloyl-β-D-glucose, caffeic acid 4-O-glucuronide and coniferyl ferulate (Fig. 5c). The abundance of 1-O-feruloyl-β-D-glucose was highest on Day 0 in both groups, declined gradually during fermentation, and slightly rebounded on Day 20 but remained significantly lower than the initial level. Coniferyl ferulate was also most abundant on Day 0 and decreased throughout fermentation. In contrast, the abundance of caffeic acid 4-O-glucuronide was lowest on Day 0 in both groups and increased significantly as lignocellulose degradation progressed. 1-O-Feruloyl-β-D-glucose is associated with plant cell wall formation. It is an ester conjugate formed from ferulic acid and glucose and is a core structural component of the lignin-carbohydrate complex (LCC) in WS (López Arnaldos et al. 2001). Its sustained decrease may indicate progressive disruption of the LCC network, suggesting that *I. lacteus* QJ can gradually depolymerize lignocellulose during fermentation. Caffeic acid 4-O-glucuronide, a common phenolic acid derivative in plants, showed an increasing trend consistent with previous reports (Qi et al. 2025), further supporting the involvement of *I. lacteus* QJ in lignocellulose degradation. Coniferyl ferulate, present in the lignin‒hemicellulose cross-linking structure of plant cell walls (Lee et al. 2017), is formed by an ester bond between coniferyl alcohol and ferulic acid. Its abundance on Day 0 was significantly greater in the IW group than in the IN group. This difference can be attributed to NaOH pretreatment disrupting short-chain ester bonds between lignin and hemicellulose in WS. During fermentation, the abundance of coniferyl ferulate decreased significantly in both groups, indicating that *I. lacteus* QJ can effectively degrade lignocellulose and disrupt the cell walls of WS.

Among benzenoids, 96 DEMs were identified in the IW group and 138 in the IN group, with 54 DEMs common to both groups (Fig. 5b). The common DEMs included ethyl gallate, methyl 6-O-galloyl-β-D-glucopyranoside, 2-O-galloyl-1,4-galactarolactone, 4-hydroxy-benzenepropanedioate, and heptyl 4-hydroxybenzoate (Fig. 5d). In both groups, the abundances of these five common DEMs were lowest on Day 0, and increased significantly during SSF. All five compounds are derivatives with gallic acid and 4-hydroxybenzoic acid as their basic structural units. Gallic acid, 4-hydroxybenzoic acid and other hydroxybenzoic acid-type phenolic acids inhibit fungal growth (Li et al. 2017). In contrast, white-rot fungi have evolved a complete aromatic compound metabolic system, which enables them to convert phenolic acids into central carbon metabolic intermediates via intracellular oxidation, decarboxylation, and ring-cleavage reactions, thereby achieving detoxification (del Cerro et al. 2021; Kato et al. 2024). Moreover, Zhao et al. (2021) reported that the degradation of lignocellulose by fungi also promotes the synthesis of certain derivatives. Based on the above mechanisms, the accumulation of the five common DEMs observed in this study may alleviate the cytotoxicity of phenolic acids to *I. lacteus* QJ and further promote lignocellulose degradation.

To identify metabolites closely associated with lignocellulose degradation, we performed Pearson correlation analyses between the common DEMs and key phenotypic indicators (crude protein content, reducing sugar content, dry matter loss, and degradation rates of cellulose, hemicellulose, and lignin). The results revealed significant correlations between these DEMs and the phenotypic traits. Among the common DEMs belonging to phenylpropanoids and polyketides, compounds such as kaempferol 3-glucuronide, methyl cinnamate, 6’’-O-malonyldaidzin, mammea B/BD, and caffeic acid 4-O-glucuronide were strongly positively correlated with the phenotypic indicators in both the IW (Fig. 5e) and IN (Fig. 5f) groups. The increased abundance of these metabolites may reflect active lignocellulose degradation by *I. lacteus* QJ. In contrast, multiflorin A and 4’-methylliquiritigenin 7-rhamnoside were significantly negatively correlated with the phenotypic indicators in both groups (Fig. 5e and f). Among the common DEMs of benzenoids, compounds such as 4-acetamidobenzoic acid and 4-hydroxy-benzenepropanedioate were positively correlated with the phenotypic indicators in both the IW (Fig. 5g) and IN (Fig. 5h) groups. The abundance of 4-acetamidobenzoic acid increased during fermentation. This compound can promote the activity of 3,4-dihydroxybenzoate decarboxylase, which catalyzes the decarboxylation of 3,4-dihydroxybenzoic acid to form catechol, thereby accelerating lignin degradation (Yoshida et al. 2010). Conversely, the concentration of dihydrocapsaicin was significantly negatively correlated with the degradation indicators. Its biosynthetic precursor contains a vanillyl moiety, which is a key structural component of lignin (Bernal et al. 1993; Fujiwake et al. 1982). Moreover, dihydrocapsaicin has been reported to be susceptible to peroxidase oxidation (Estrada et al. 2000). The peroxidases secreted by *I. lacteus* QJ during lignocellulose degradation may oxidize dihydrocapsaicin, thereby decreasing its abundance. In summary, these common DEMs between the two groups may serve as potential biomarkers associated with lignocellulose degradation by *I. lacteus* QJ.

#### Lignin units of WS and degradative metabolic patterns

**Fig. 6:**
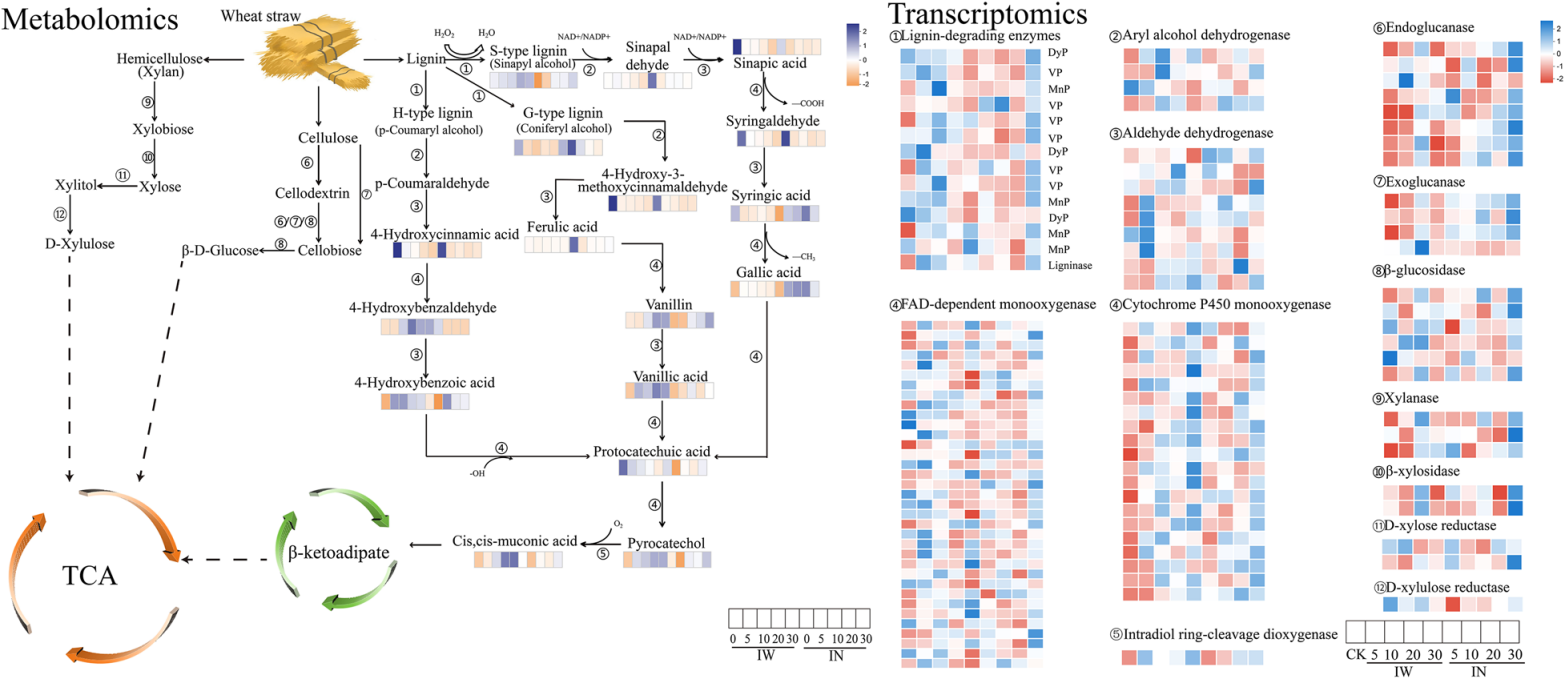
Integrated transcriptomic and metabolomic analyses investigating the mechanism of WS lignocellulose degradation by *I. lacteus* QJ*.* The left sub-figure shows the metabolomic map of the lignocellulose degradation pathway, with heatmaps next to each metabolite showing the changes in their relative abundance during 0–30 days of fermentation in the IW and IN groups; The right sub-figure shows the transcriptomic expression map of key lignocellulolytic enzymes, with the heatmaps presenting the relative expression levels of the genes encoding 12 core lignocellulolytic enzymes during the same fermentation period. These 12 enzymes catalyze the key steps involved in lignocellulose degradation. In the metabolomic heatmap, metabolite abundance is indicated by color intensity where deep blue represents high abundance and lighter red indicates lower abundance. In the transcriptomic analysis, gene expression levels are color-coded where darker blue denotes higher expression levels and lighter red denotes lower expression levels

Lignin typically consists of three types (S-type, G-type, and H-type), whose corresponding basic units are the syringyl unit (sinapyl alcohol), guaiacyl unit (coniferyl alcohol), and p-hydroxyphenyl unit (p-coumaryl alcohol), respectively. Figure 6 shows that only the abundances of G-type (coniferyl alcohol) and S-type (sinapyl alcohol) lignin units were detected on Day 0, whereas those of the H-type (p-coumaryl alcohol) were not. This finding indicates that WS lignin is rich in G-type and S-type units but scarce in H-type units, which is consistent with the previously reported composition characteristics of WS lignin (Zeng et al. 2013). Although neither H-type lignin nor its subsequent metabolite p-coumaraldehyde was detected in the metabolomic analysis, their related intermediate metabolites, such as 4-hydroxybenzaldehyde and 4-hydroxybenzoic acid, were identified, confirming the presence of low-abundance H-type lignin in WS.

NaOH pretreatment markedly altered the abundance profiles of key metabolites involved in lignin biosynthesis. Compared with the raw WS, in the S-type lignin pathway, sinapyl alcohol decreased to 0.8942 of the raw level, syringaldehyde increased to 1.0234, and syringic acid decreased to 0.7819. In the G-type pathway, the level of ferulic acid increased to 1.1557, while that of vanillin decreased to 0.9230. In the H-type pathway, 4-hydroxybenzaldehyde increased to 1.2738. The key lignin degradation intermediate, protocatechuic acid, decreased to 0.9552 (Supplementary Table S9). OH⁻ generated by NaOH dissociation can specifically cleave the ester bonds between ferulic acid and hemicellulose within the lignin-carbohydrate complex, irreversibly disrupting the lignin–hemicellulose crosslinking network and thereby dismantling the recalcitrance barrier of lignocellulose (Modenbach and Nokes 2014; Buranov and Mazza 2008). This mechanism is highly consistent with our observation of a marked increase in the abundance of ferulic acid after NaOH pretreatment: the hydrolysis of ester bonds releases large amounts of ferulic acid originally bound to hemicellulose, thereby increasing its content (Ideia et al. 2020). Despite these differences, common lignin degradation patterns were detected in both groups. The abundances of most upstream metabolites gradually decreased as fermentation progressed, while downstream metabolites accumulated continuously. This indicates that *I. lacteus* QJ exhibits similar degradation patterns for different lignin types. For example, the abundances of upstream metabolites, including sinapaldehyde, sinapic acid, syringaldehyde, coniferyl alcohol, vanillin, and 4-hydroxycinnamic acid, were greatest in both groups on Day 0 and tended to decrease as fermentation progressed. In contrast, downstream metabolites such as gallic acid, vanillic acid, and 4-hydroxybenzoic acid accumulated progressively throughout fermentation (Supplementary Table S9).

### Integrated transcriptomic and metabolomic analyses

This study systematically investigated the mechanism of WS lignocellulose degradation by *I. lacteus* QJ using integrated transcriptomic and metabolomic analyses (Fig. 6). With respect to lignin degradation, the process can be divided into two stages: depolymerization and mineralization. During depolymerization, *I. lacteus* QJ upregulates the expression of lignin-degrading enzymes such as VP, MnP, and DyP. These enzymes break the covalent bonds within lignin, facilitating reactions such as C–C and C-O bond cleavage and depolymerizing lignin into simple monomers such as sinapyl alcohol, coniferyl alcohol, and p-coumaryl alcohol (Kumar et al. 2021). In the subsequent mineralization stage, these monomers are oxidized to corresponding aldehydes by aryl alcohol dehydrogenase and then further oxidized to sinapic acid, ferulic acid, and 4-hydroxycinnamic acid by aldehyde dehydrogenase. These phenolic acids are subsequently converted to syringaldehyde, vanillin, and 4-hydroxybenzaldehyde through redox reactions and then further oxidized to syringic acid, vanillic acid, and 4-hydroxybenzoic acid by decarboxylation. Flavin monooxygenase and CYP450 enzymes subsequently mediate hydroxylation and demethylation reactions, resulting in the formation of gallic acid, protocatechuic acid, and protocatechol (Erickson et al. 2022). Protocatechol ring cleavage is determined by intradiol and extradiol ring-cleaving dioxygenases. Intradiol ring-cleaving dioxygenase catalyzes the intradiol cleavage of catechol to *cis,cis*-muconic acid, a crucial step for aromatic compounds entering microbial metabolism during polycyclic aromatic hydrocarbon degradation, laying the foundation for subsequent complete mineralization (Vaillancourt et al. 2006). Consistent with the transcriptomic results, *I. lacteus* QJ promotes lignin degradation by upregulating the expression of intradiol ring-cleaving dioxygenase genes. Ultimately, *cis,cis*-muconic acid formed by intradiol cleavage enters the TCA cycle for complete metabolism via the β-ketoadipate pathway (Brink et al. 2019; Zhu et al. 2022). Cellulose degradation mainly involves the conversion of cellodextrin, cellobiose, and β-D-glucose, with the significant upregulation of the expression of genes encoding related enzymes, such as endoglucanase, exoglucanase, and β-glucosidase, during fermentation. Hemicellulose degradation is dominated by xylan breakdown, with degradation products including xylobiose, xylose xylitol, and D-xylulose, involving key enzymes such as xylanase, β-xylosidase, D-xylitol reductase, and D-xylulose reductase.

In summary, when degrading both raw WS and NaOH-pretreated WS, *I. lacteus* QJ maintains stable core degradation pathways, with most metabolites showing similar changes and upregulated expression of genes encoding degrading enzymes. Moreover, NaOH pretreatment significantly alters the abundance of some metabolites (e.g., 4-hydroxybenzaldehyde and syringic acid), which may indirectly affect the efficiency of lignin degradation by *I. lacteus* QJ.

## Conclusion

This study revealed that *I. lacteus* QJ effectively degraded raw and NaOH-pretreated WS through SSF. *I. lacteus* QJ significantly reduced lignocellulose content and increased crude protein and reducing sugars, confirming its ability to both degrade lignocellulose and convert nutrients. *I. lacteus* QJ degrades lignocellulose both by secreting a variety of lignocellulolytic enzymes that cleave chemical bonds and reduce straw crystallinity and by physically disrupting the structure of the straw with its hyphae. Integrated multi-omics analyses revealed that the DEGs in the two groups were mainly involved in oxidoreductase activity, monooxygenase activity, cellulose catabolism, glucan catabolism and amino acid metabolism, which fully demonstrated the capacity of *I. lacteus* QJ to efficiently degrade lignocellulose and promote nutrient bioconversion. Key enzymes (e.g., phenol hydroxylase, aldehyde dehydrogenase and D-xylulose reductase) played crucial roles in lignocellulose degradation in both groups. Although NaOH pretreatment caused a significant alteration in the metabolite abundance of WS, *I. lacteus* QJ exhibited a consistent pattern of lignocellulose degradation between the two groups. Furthermore, metabolites, including caffeic acid 4-O-glucuronide, 4-acetamidobenzoic acid and methyl cinnamate, were significantly positively correlated with the lignocellulose degradation of *I. lacteus* QJ. These results provide a mechanistic foundation for understanding *I. lacteus* QJ-mediated straw degradation and support its potential application in straw resource utilization.

## Supplementary Information

Below is the link to the electronic supplementary material.

Supplementary Material 1

## Data Availability

The raw transcriptome sequencing data and raw metabolome sequencing data generated in this study have been deposited in the NCBI database (accession number: PRJNA1328065) and the MetaboLights database (accession number: MTBLS13099), respectively. All additional data supporting the validation of the conclusions of this paper are available in the main text and Supplementary Material.
