# Supplementary material for: Lignocellulose degradation characteristics and mechanisms of raw and NaOH-pretreated wheat straw by Irpex lacteus QJ: multi-omics analysis
Source: Bioresour Bioprocess. 2026 Jul 10;13(1):100. doi: 10.1186/s40643-026-01091-8 (PMC13354742; doi:10.1186/s40643-026-01091-8)
Supplement: Supplementary file 1 — Supplementary Material 1 [file 40643_2026_1091_MOESM1_ESM.docx]

**Table S1**

Information of qPCR primer sequences, and gene expression levels by transcriptomic sequencing.

| **Gene ID** | **Primer sequence** | **Description** | **Expression level calculated by the TMP reads method** | | | | | | | | |
| --- | --- | --- | --- | --- | --- | --- | --- | --- | --- | --- | --- |
|  |  |  | **CK** | **IW(d)** | | | | **IN(d)** | | | |
|  |  |  |  | **5** | **10** | **20** | **30** | **5** | **10** | **20** | **30** |
| DN1408_c0_g1 | Forward: GCAAGAACTTATCGCCTTCG | Phenol hydroxylase | 7.43 | 9.34 | 28.41 | 32.56 | 11.31 | 33.16 | 23.86 | 22.61 | 117.31 |
|  | Reverse: GAAGTGAGCCCACTGAAAGC |  |  |  |  |  |  |  |  |  |  |
| DN159_c0_g1 | Forward: GGAAAACAACTCGCCCTACA | Endo-1,4-beta-xylanase | 68.45 | 77.31 | 168.57 | 173.63 | 256.42 | 78.67 | 121.77 | 148.86 | 196.14 |
|  | Reverse: ACGAGTCCAACCAGTGAACC |  |  |  |  |  |  |  |  |  |  |
| DN166_c1_g2 | Forward: CTGACAAGGCCAAGACAACA | D-xylulose reductase | 35.16 | 99.52 | 165.84 | 116.92 | 125.63 | 74.43 | 82.78 | 167.03 | 131.30 |
|  | Reverse: GCACTCGAAGACAACGTCAA |  |  |  |  |  |  |  |  |  |  |
| DN1723_c3_g1 | Forward: ACTGCTTCAAGGACGCAACT | Catalase-1 | 4.08 | 63.27 | 26.08 | 207.97 | 232.12 | 35.67 | 9.33 | 123.00 | 27.62 |
|  | Reverse: TGGTAGTTCATGACGGGACA |  |  |  |  |  |  |  |  |  |  |
| DN1960_c0_g3 | Forward: TACCGTCGCTGCTCAGTATG | Versatile peroxidase VPS1 | 5.47 | 184.54 | 736.66 | 154.94 | 268.53 | 49.94 | 21.49 | 57.61 | 696.38 |
|  | Reverse: CGTTGCCAGTGAAAGCAGTA |  |  |  |  |  |  |  |  |  |  |
| DN2900_c0_g1 | Forward: GGTTCGTAAGGCGTTGGATA | Phenol hydroxylase | 13.63 | 78.42 | 60.93 | 39.11 | 127.20 | 8.04 | 153.33 | 133.80 | 67.13 |
|  | Reverse: TGGACTTGAGAAGTGCAACG |  |  |  |  |  |  |  |  |  |  |
| DN294_c0_g2 | Forward: CAAAACTGGGACGCCACTAT | Aldehyde dehydrogenase | 3.60 | 227.08 | 12.94 | 16.50 | 17.25 | 34.85 | 157.41 | 33.92 | 51.09 |
|  | Reverse: AGGAAGCAAGGTCAAGACGA |  |  |  |  |  |  |  |  |  |  |
| DN1648_c0_g1 | Forward: CCGTATGGTGTCAACAGTGC | Gamma tubulin | 114.29 | 185.93 | 124.56 | 135.35 | 149.77 | 127.73 | 124.06 | 120.72 | 146.64 |
|  | Reverse: CGTCGGTAGGGTCAACATCT |  |  |  |  |  |  |  |  |  |  |

**
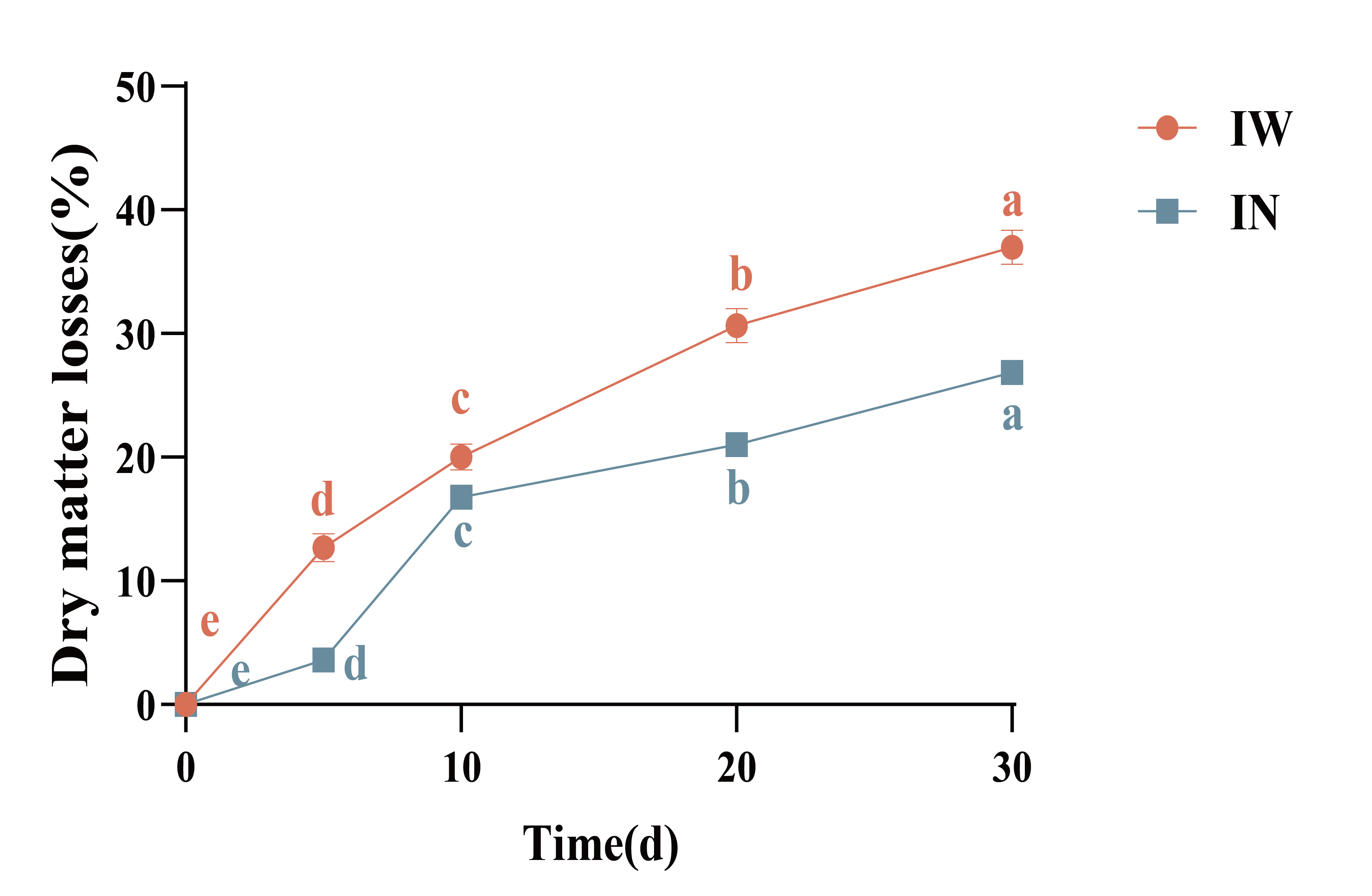
**

**Fig. S1** Dry matter loss rates of WS in the IW and IN groups during SSF. Different lowercase letters in the same group indicate significant differences (*P* < 0.05).

**
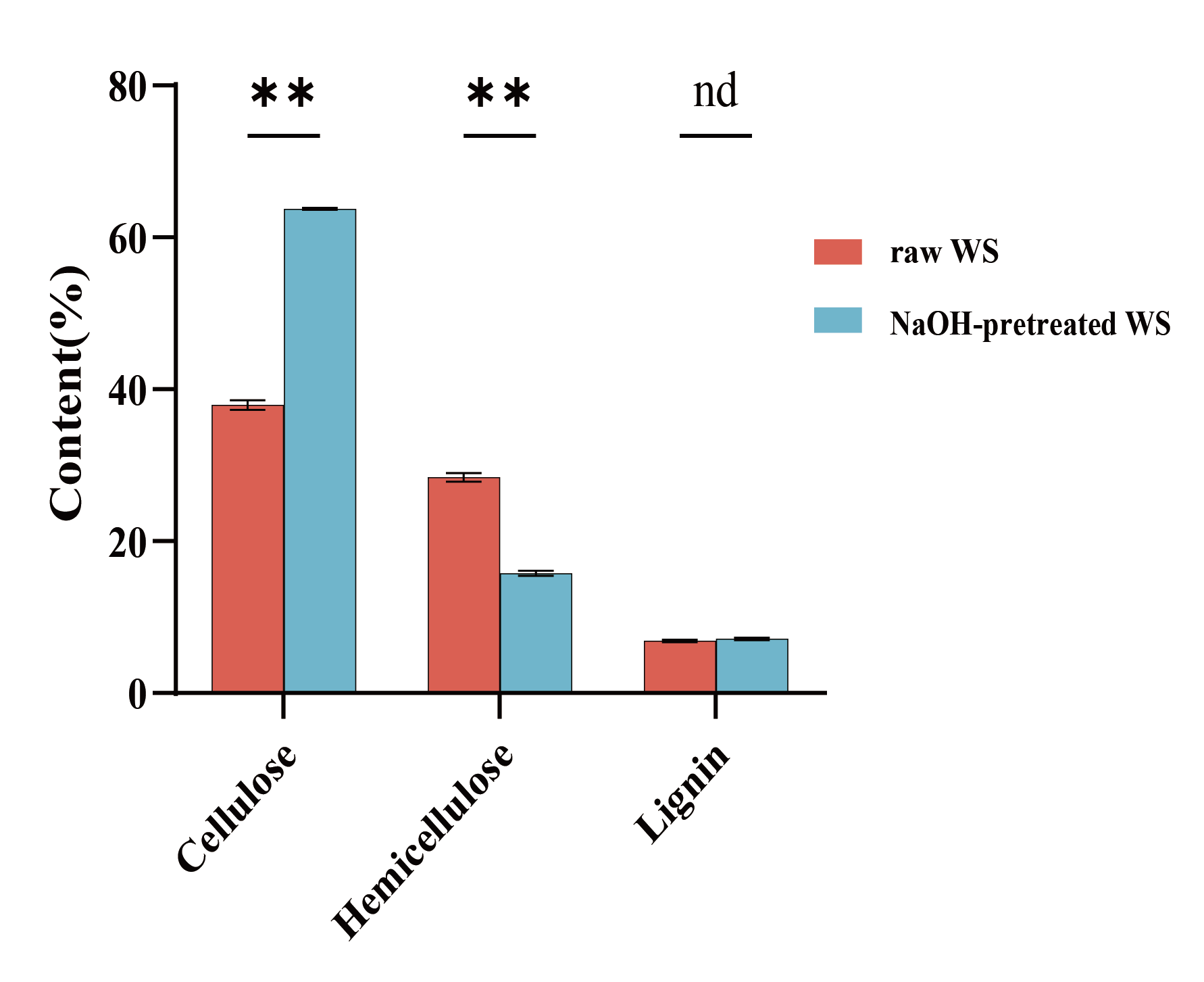
**

**Fig. S2** Comparison of lignocellulose content between raw WS and NaOH-pretreated WS. “**” means significant difference at *P* < 0.01, and “nd” means no significant difference.

**
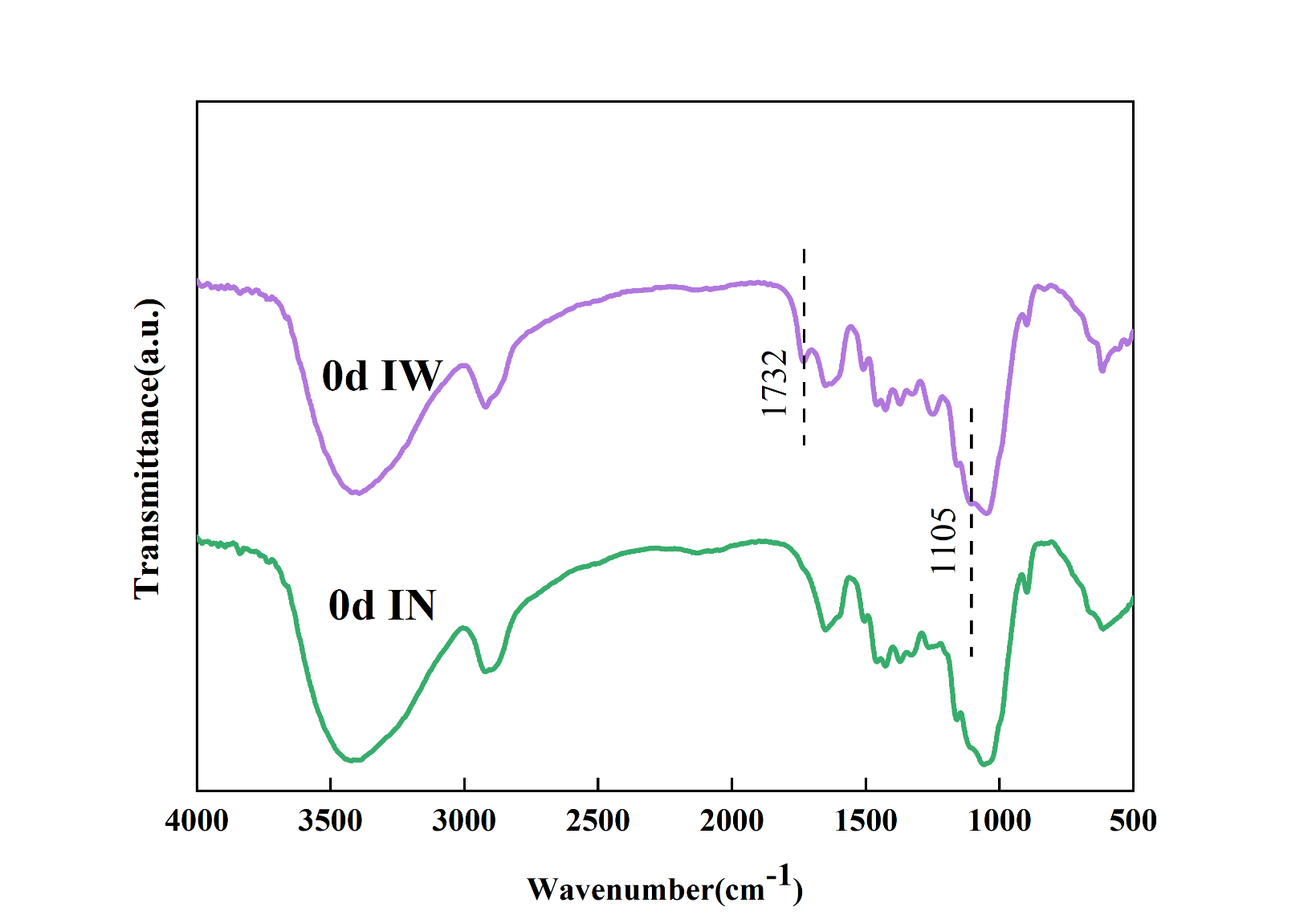
**

**Fig. S3** FTIR spectra of WS in groups IW and IN on day 0.

**Table S2**

FTIR spectral assignments and relative peak intensities for the lignocellulose of the IW group.

| **Wave number**  **(cm^-1^)** | **Assignment** | **Biomass component** | **Intensity (% relative percentage changes)** | | | |
| --- | --- | --- | --- | --- | --- | --- |
|  |  |  | **5d** | **10d** | **20d** | **30d** |
| 3394 | O-H stretching | Cellulose | 23.54 | 43.88 | 9.07 | 26.44 |
| 2922 | C-H stretching | Cellulose, hemicellulose | 15.53 | 18.77 | -7.42 | 6.63 |
| 1732 | C=O acetyl group | Hemicellulose | 5.02 | 2.73 | -17.98 | -7.74 |
| 1651 | C=C stretching | Lignin | -0.58 | 6.61 | -19.63 | -8.88 |
| 1157 | C-O-C asymmetric stretching | Cellulose, hemicellulose | 25.74 | 36.90 | **/** | **/** |
| 1047 | C-O telescopic vibration | Cellulose, hemicellulose | 38.00 | 62.21 | 16.17 | 34.93 |
| 899 | β (1,4) glycosidic bond | Cellulose amorphous | 4.57 | 2.94 | -6.70 | 2.49 |

Positive values indicated bond cleavage, while negative values indicated no change.

**Table S3**

FTIR spectral assignments and relative peak intensities for the lignocellulose of the IN group.

| **Wave number**  **(cm^-1^)** | **Assignment** | **Biomass component** | **Intensity (% relative percentage changes)** | | | |
| --- | --- | --- | --- | --- | --- | --- |
|  |  |  | **5d** | **10d** | **20d** | **30d** |
| 3421 | O-H stretching | Cellulose | -4.95 | 20.33 | -25.10 | 53.99 |
| 2924 | C-H stretching | Cellulose, hemicellulose | -9.07 | 8.98 | -14.75 | 21.16 |
| 1650 | C=C stretching | Lignin | -14.03 | -3.45 | -23.48 | 4.56 |
| 1159 | C-O-C asymmetric stretching | Cellulose, hemicellulose | -10.75 | 11.86 | -20.39 | 31.00 |
| 1059 | C-O telescopic vibration | Cellulose, hemicellulose | -7.07 | 21.58 | -23.86 | 50.81 |
| 897 | β (1,4) glycosidic bond | Cellulose amorphous | -3.34 | 3.29 | -1.71 | 7.44 |

Positive values indicated bond cleavage, while negative values indicated no change.

**Table S4**

Quality control statistics of the transcriptomic sequencing data from *I. lacteus.*

| **Sample** | **Raw reads** | **Raw bases** | **Clean reads** | **Clean bases** | **Q20**  **(%)** | **Q30**  **(%)** |
| --- | --- | --- | --- | --- | --- | --- |
| CK1 | 55086214 | 8318018314 | 54456976 | 8118528113 | 98.75 | 95.91 |
| CK1 | 55086214 | 8318018314 | 54456976 | 8118528113 | 98.75 | 95.91 |
| CK2 | 61083020 | 9223536020 | 60512040 | 9049453558 | 98.75 | 95.9 |
| CK3 | 58084364 | 8770738964 | 57420762 | 8574820323 | 98.75 | 95.91 |
| IW-5d-1 | 66421536 | 10029651936 | 65743118 | 9810906832 | 98.79 | 96.04 |
| IW-5d-2 | 58811730 | 8880571230 | 58217298 | 8727597015 | 98.73 | 95.86 |
| IW-5d-3 | 55085552 | 8317918352 | 54538290 | 8112290062 | 98.74 | 95.89 |
| IW-10d-1 | 64888234 | 9798123334 | 64284058 | 9615164122 | 98.74 | 95.88 |
| IW-10d-2 | 61605594 | 9302444694 | 60991070 | 9103859442 | 98.75 | 95.9 |
| IW-10d-3 | 65563172 | 9900038972 | 64896338 | 9691991855 | 98.73 | 95.84 |
| IW-20d-1 | 64948872 | 9807279672 | 64281088 | 9578677758 | 98.78 | 95.99 |
| IW-20d-2 | 58148822 | 8780472122 | 57611468 | 8599224622 | 98.68 | 95.67 |
| IW-20d-3 | 53455942 | 8071847242 | 52945512 | 7916284042 | 98.76 | 95.93 |
| IW-30d-1 | 57060326 | 8616109226 | 56523814 | 8454247121 | 98.77 | 95.97 |
| IW-30d-2 | 60746990 | 9172795490 | 60106762 | 8942420631 | 98.78 | 96 |
| IW-30d-3 | 58085930 | 8770975430 | 57358516 | 8514598041 | 98.73 | 95.85 |
| IN-5d-1 | 53336398 | 8053796098 | 52769556 | 7918279279 | 98.74 | 95.88 |
| IN-5d-2 | 63704058 | 9619312758 | 63087702 | 9442901426 | 98.76 | 95.94 |
| IN-5d-3 | 58887202 | 8891967502 | 58317814 | 8716360619 | 98.74 | 95.86 |
| IN-10d-1 | 57161050 | 8631318550 | 56584396 | 8448297446 | 98.76 | 95.94 |
| IN-10d-2 | 61800500 | 9331875500 | 61206510 | 9156286113 | 98.75 | 95.89 |
| IN-10d-3 | 59793082 | 9028755382 | 59190124 | 8848719803 | 98.78 | 95.99 |
| IN-20d-1 | 48380064 | 7305389664 | 47877818 | 7185495884 | 98.72 | 95.82 |
| IN-20d-2 | 59193754 | 8938256854 | 58542962 | 8744935656 | 98.67 | 95.65 |
| IN-20d-3 | 52962178 | 7997288878 | 52399382 | 7842397115 | 98.7 | 95.75 |
| IN-30d-1 | 58002556 | 8758385956 | 57450592 | 8587239899 | 98.75 | 95.93 |
| IN-30d-2 | 60830966 | 9185475866 | 60019832 | 8907674358 | 98.76 | 95.95 |
| IN-30d-3 | 61285464 | 9254105064 | 60608208 | 9029397384 | 98.73 | 95.86 |

**Table S5**

Summary of unigenes and transcripts from the *I. lacteus* transcriptome.

| **Type** | **Unigene** | **Transcript** |
| --- | --- | --- |
| Total number | 9921 | 45744 |
| Total base | 26333700 | 162032092 |
| Largest length (bp) | 19870 | 19870 |
| Smallest length (bp) | 201 | 201 |
| Average length (bp) | 2654.34 | 3542.15 |
| N50 length (bp) | 3953 | 5001 |
| E90N50 length (bp) | 4123 | 3740 |
| Fragment mapped percent (%) | 80.327 | 89.769 |
| GC percent (%) | 51.98 | 51.68 |
| TransRate score | 0.34841 | 0.40143 |
| BUSCO score | C:83.7% [S:83.4%; D:0.3%] | C:93.7% [S:40.3%; D:53.4%] |


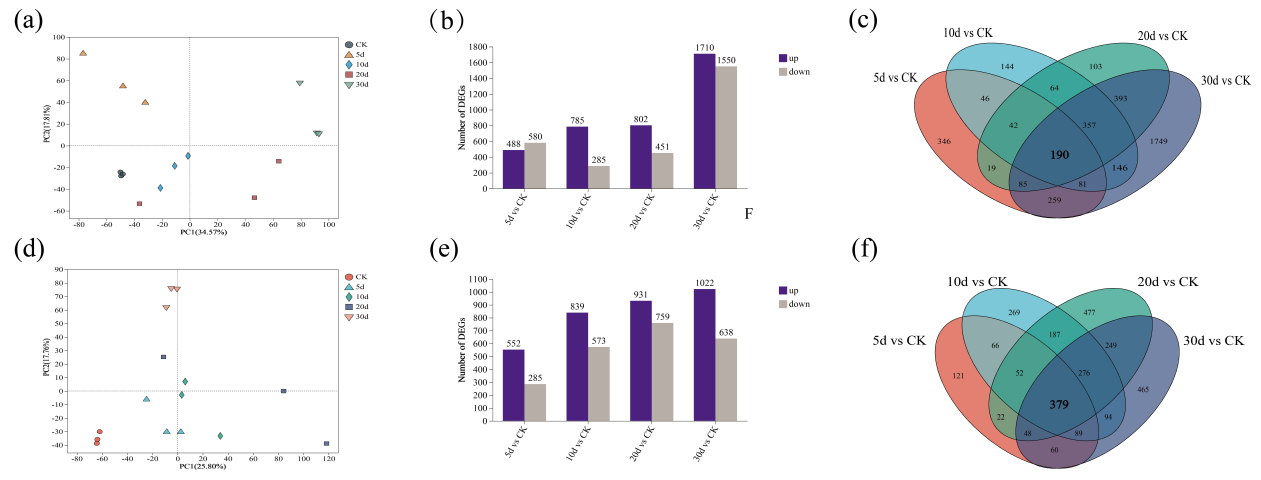


**Fig. S4** Transcriptomic profiling of *I. lacteus* in the IW and IN groups. (a) PCA plot of the IW group. (b) Number of DEGs in the IW group. (c) Venn diagram of DEGs in the IW group. (d) PCA plot of the IN group. (e) Number of DEGs in the IN group. (f) Venn diagram of DEGs in the IN group. All DEGs were screened based on the criteria of *P* < 0.05 and |log₂(FC)| ≥ 1.


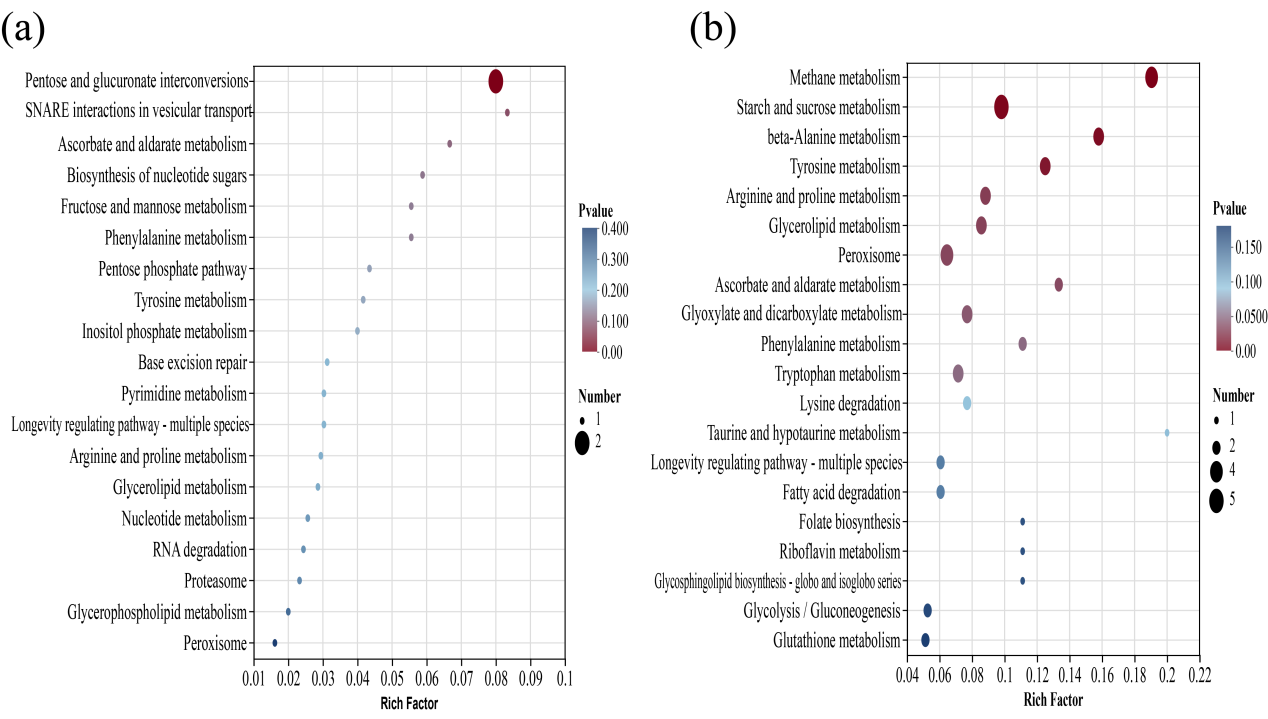


**Fig. S5** KEGG enrichment analysis of common upregulated DEGs in the IW (a) and the IN (b) group. The pathways were enriched under the condition of *P* < 0.5.

**Table S6**

The relative expression level of the *prx1* gene during fermentation in both groups.

| **Gene id** | **KO name** | **Expression level calculated by the TMP reads method** | | | | | | | | |
| --- | --- | --- | --- | --- | --- | --- | --- | --- | --- | --- |
|  |  | **CK** | **IW(d)** | | | | **IN(d)** | | | |
|  |  |  | **5** | **10** | **20** | **30** | **5** | **10** | **20** | **30** |
| DN4459_c0_g2 | *prx1* | 2728.52 | 280.28 | 609.17 | 778.60 | 366.27 | 665.95 | 685.68 | 574.58 | 771.15 |

**Table S7**

NR and Swiss-Prot annotation of the core proteins identified through PPI analysis in the IW group.

| **Protein Name** | **Gene ID** | **NR** | **Swiss-Prot** |
| --- | --- | --- | --- |
| JGI70824 | DN2900_c0_g1 | FAD binding domain-containing protein | Phenol hydroxylase |
| JGI72977 | DN310_c1_g2 | Monooxygenase | Flavin-dependent monooxygenase |
| JGI78627 | DN166_c1_g2 | Chaperonin 10-like protein | Probable D-xylulose reductase A |
| JGI23655 | DN1352_c1_g1 | Glycerol kinase | Glycerol kinase |
| JGI63757 | DN256_c1_g2 | DAO-domain-containing protein | Glycerol-3-phosphate dehydrogenase, mitochondrial |
| JGI22987 | DN2108_c0_g1 | Common central domain of tyrosinase-domain-containing protein | Tyrosinase |
| JGI75638 | DN1352_c1_g1 | Glycerol kinase | Glycerol kinase |
| JGI78981 | DN294_c1_g1 | Chaperonin 10-like protein | Probable D-xylulose reductase A |
| PcCYP_142c | DN3412_c0_g1 | Cytochrome P450 | Cytochrome P450 monooxygenase 205 |
| JGI29155 | DN448_c0_g1 | Cytochrome P450 | Cytochrome P450 monooxygenase 75 |
| PcCYP_1c | DN291_c0_g1 | Putative CyP450 monooxygenase | Cytochrome P450 monooxygenase COX2 |
| JGI94003 | DN81_c0_g1 | Cytochrome P450 | Cytochrome P450 monooxygenase 58 |
| JGI96083 | DN81_c1_g1 | Cytochrome P450 | Cytochrome P450 monooxygenase 58 |
| A2.12f | DN952_c1_g2 | Not1 N-terminal domain, CCR4-Not complex component-domain-containing protein | General negative regulator of transcription subunit 3 |
| JGI63489 | DN2691_c0_g1 | Hypothetical protein BC629DRAFT_1595604 | Superoxide dismutase [Cu-Zn] |
| JGI71076 | DN1277_c0_g1 | NAD(P)-binding protein | D-arabinitol 2-dehydrogenase |
| JGI96410 | DN185_c1_g1 | Chaperonin 10-like protein | Probable D-xylulose reductase A |
| JGI74598 | DN500_c0_g1 | Hypothetical protein BC629DRAFT_1535921, partial | NADP-dependent 3-hydroxy acid dehydrogenase |

**Table S8**

NR and Swiss-Prot annotation of the core proteins identified through PPI analysis in the IN group.

| **Protein Name** | **Gene ID** | **NR** | **Swiss-Prot** |
| --- | --- | --- | --- |
| JGI94094 | DN294_c0_g2 | Aldehyde dehydrogenase | Aldehyde dehydrogenase, dimeric NADP-preferring |
| JGI70824 | DN1408_c0_g1 | FAD binding domain-containing protein | Phenol hydroxylase |
| JGI99197 | DN1723_c3_g1 | Catalase | Catalase-1 |
| JGI78627 | DN166_c1_g2 | Chaperonin 10-like protein | Probable D-xylulose reductase A |
| JGI94779 | DN3718_c0_g1 | S-(hydroxymethyl) glutathione dehydrogenase | S; |
| JGI69457 | DN342_c0_g1 | Hypothetical protein BC629DRAFT_1280122 | Formate dehydrogenase |
| JGI93978 | DN300_c0_g3 | NAD-dependent formate dehydrogenase | Formate dehydrogenase |
| JGI67082 | DN13_c0_g1 | Aminotransferase | Probable aspartate/prephenate aminotransferase |
| JGI78981 | DN294_c1_g1 | Hypothetical protein BC629DRAFT_1587199 | Uncharacterized oxidoreductase C2F3.05c |
| CPR | DN3367_c1_g1 | Cytochrome P450 oxidoreductase | NADPH-cytochrome P450 reductase |
| JGI76966 | DN858_c0_g1 | L-lactate dehydrogenase | FMN-dependent alpha-hydroxy acid dehydrogenase qulF |
| JGI70076 | DN1695_c0_g1 | Agmatinase | Cytochrome P450 monooxygenase 88 |
| JGI22987 | DN2108_c0_g1 | Common central domain of tyrosinase-domain-containing protein | Tyrosinase |
| JGI29195 | DN844_c1_g1 | Ornithine aminotransferase | Ornithine aminotransferase car2 |
| PcCYP_81c | DN771_c0_g1 | Cytochrome P450 | Cytochrome P450 monooxygenase 124 |
| JGI63489 | DN2691_c0_g1 | Hypothetical protein BC629DRAFT_1595604 | Superoxide dismutase [Cu-Zn] |
| JGI63410 | DN2944_c0_g1 | Hydroxymethylglutaryl-coenzyme A reductase-domain-containing protein | 3-Hydroxy-3-methylglutaryl-coenzyme A reductase |
| JGI29155 | DN448_c0_g1 | Cytochrome P450 | Cytochrome P450 monooxygenase 75 |
| JGI71076 | DN1277_c0_g1 | NAD(P)-binding protein | D-arabinitol 2-dehydrogenase |
| JGI96410 | DN185_c1_g1 | Hypothetical protein BC629DRAFT_1455850 | Short-chain dehydrogenase/reductase ARMGADRAFT_1018421 |
| JGI23655 | DN1352_c1_g1 | Glycerol kinase | Glycerol kinase |
| JGI98431 | DN1749_c0_g2 | Glutamate decarboxylase | Glutamate decarboxylase |
| JGI96286 | DN544_c1_g2 | Saccharopine dehydrogenase-domain-containing protein | Alpha-aminoadipic semialdehyde synthase, mitochondrial |
| JGI70645 | DN1096_c0_g1 | S-adenosyl-L-methionine-dependent methyltransferase | Uncharacterized methyltransferase C1B3.06c |
| JGI68730 | DN238_c3_g3 | GroES-like protein | Trans-enoyl reductase ACTTS2 |
| PcCYP_1a | DN326_c1_g1 | Cytochrome P450 | Cytochrome P450 monooxygenase 89 |
| JGI74598 | DN500_c0_g1 | Hypothetical protein BC629DRAFT_1535921 | NADP-dependent 3-hydroxy acid dehydrogenase |


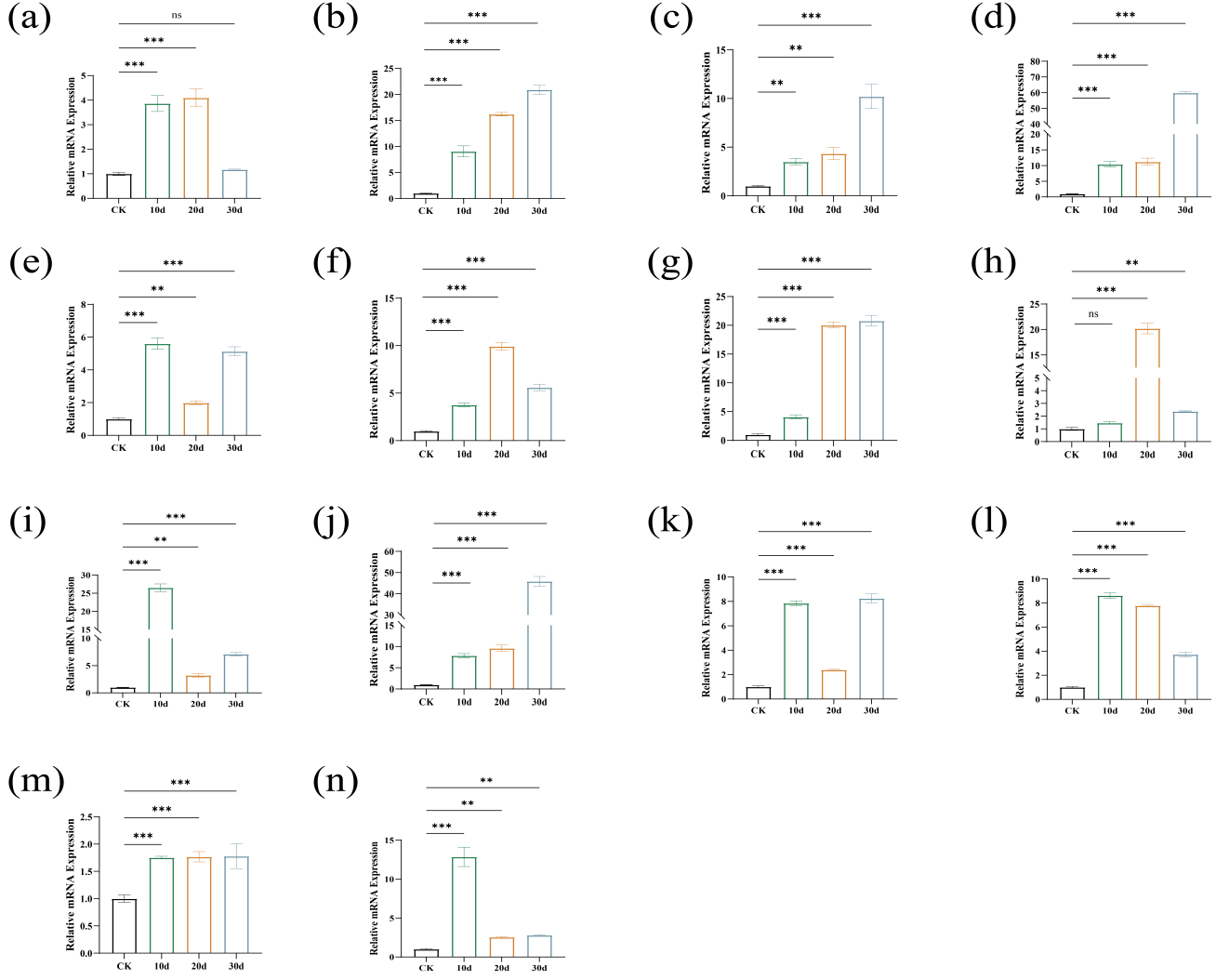


**Fig. S6** Relative mRNA expression of seven lignocellulose-degrading genes in *I. lacteus* from the IW and IN groups. (a–b) DN1408_c0_g1 in the IW and IN groups, respectively; (c–d) DN159_c0_g1 in the IW and IN groups, respectively; (e–f) DN166_c1_g2 in the IW and IN groups, respectively; (g–h) DN1723_c3_g1 in the IW and IN groups, respectively; (i–j) DN1960_c0_g3 in the IW and IN groups, respectively; (k–l) DN2900_c0_g1 in the IW and IN groups, respectively; (m–n) DN294_c0_g2 in the IW and IN groups, respectively. “***” and “**” indicate significant differences at *P* < 0.01 and *P* < 0.05, respectively; “ns” indicates no significant difference.


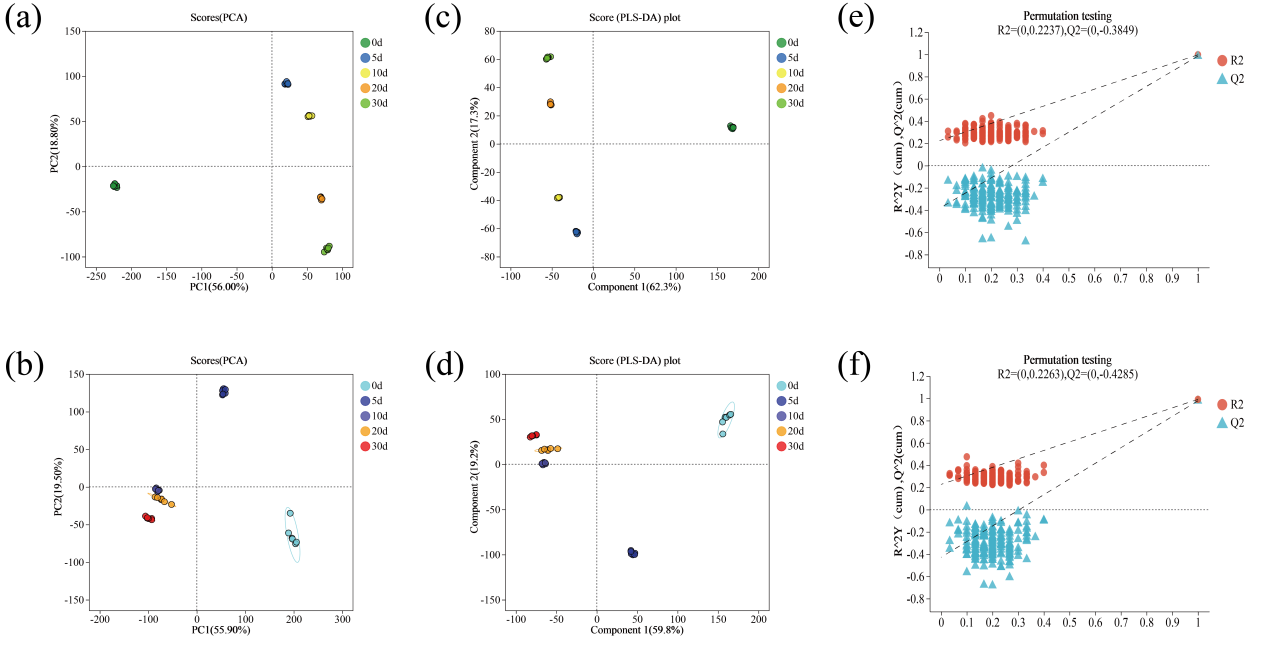


**Fig. S7** Statistical analysis of metabolomics data. (a–b) PCA plots for the IW and IN groups, respectively. (c–d) PLS-DA plots for the IW and IN groups, respectively. (e–f) Permutation test plots for the IW and IN groups, respectively.


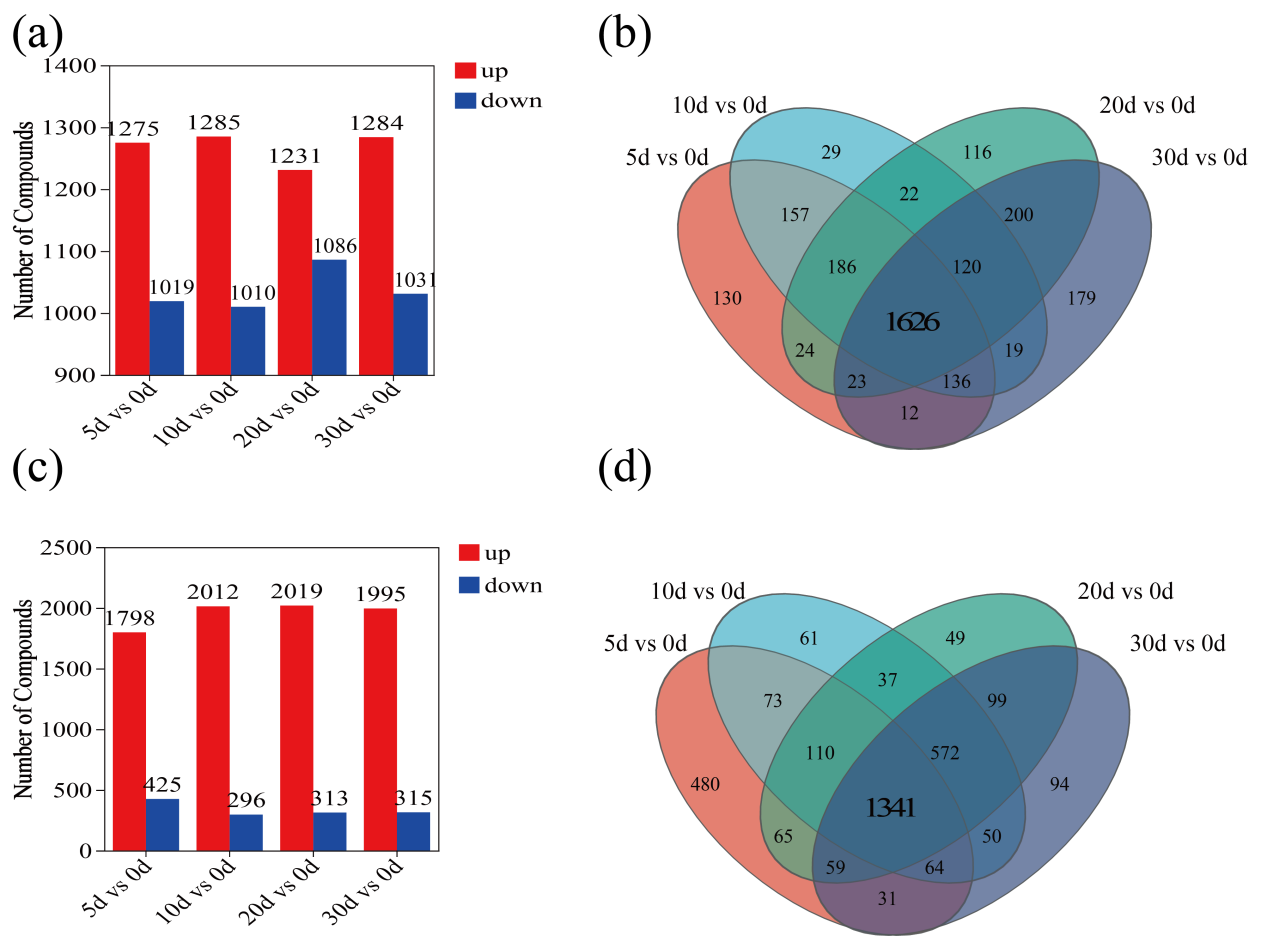


**Fig. S8** Statistical analysis of DEMs. (a, c) Numbers of DEMs in the IW (a) and IN (c) groups during SSF compared to the control. (b, d) Venn diagrams for the IW (b) and IN (d) groups, respectively. DEMs were selected using the criteria of VIP > 1 and *P* < 0.05.


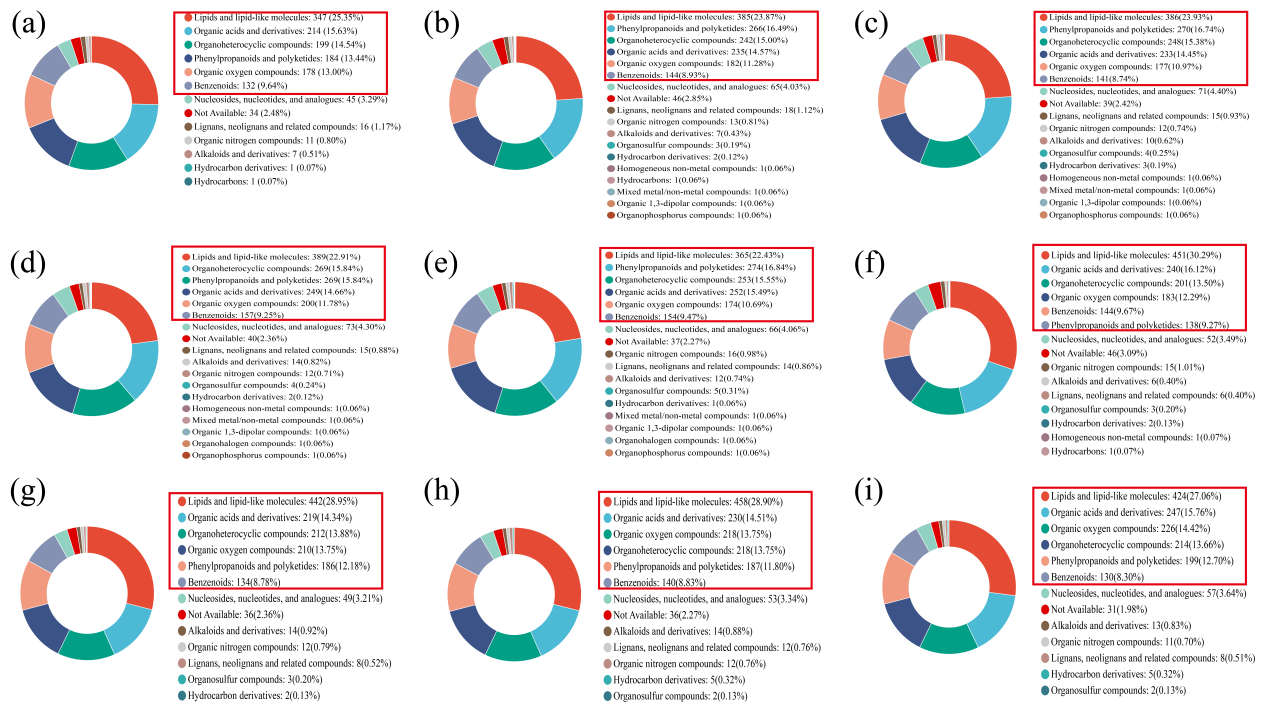


**Fig. S9** Classification of DEMs based on HMDB compound categories. (a) DEMs between the IN group at day 0 versus the IW group at day 0. (b–e) DEMs in the IW group at fermentation days 5, 10, 20, and 30, respectively. (f–i) DEMs in the IN group at fermentation days 5, 10, 20, and 30, respectively.

**Table S9**

Relative expression levels and differential fold changes of lignin-related metabolites.

| **Metabolites** | **Relative expression levels** | | | | | | | | | | **Fold Change IN(0d)/IW(0d)** |
| --- | --- | --- | --- | --- | --- | --- | --- | --- | --- | --- | --- |
|  | **IW(d)** | | | | | **IN(d)** | | | | |  |
|  | **0** | **5** | **10** | **20** | **30** | **0** | **5** | **10** | **20** | **30** |  |
| 4-Hydroxy-3-methoxycinnamaldehyde | 2.3365 | -0.3435 | -0.3766 | -0.4731 | 0.6839 | 1.5098 | -0.2253 | 0.4301 | -0.6777 | -0.6362 | 0.9385 |
| 4-Hydroxybenzoic acid | -1.4835 | 0.8903 | -0.6130 | 0.4231 | 0.3272 | -0.4396 | -1.9574 | 0.9890 | 0.1954 | 0.1436 | 1.0482 |
| 4-Hydroxybenzaldehyde | -0.5849 | -0.6216 | 0.4247 | 1.2423 | 0.7709 | 0.7765 | 0.347 | -0.746 | -0.783 | -0.8259 | 1.2738 |
| 4-Hydroxycinnamic acid | 1.8044 | 0.1083 | -0.1001 | -0.5924 | 0.8693 | 1.5966 | -0.5405 | -0.3601 | -0.5741 | -0.4727 | 0.9785 |
| *Cis,cis*-muconic acid | -1.3299 | -0.4827 | 0.4222 | 1.5330 | 1.6317 | 0.0102 | -1.4426 | -0.1345 | -0.4374 | 0.2299 | 1.0326 |
| Coniferyl alcohol | 0.9418 | -0.8784 | -1.2224 | -0.6568 | -0.8772 | 0.8738 | 1.9691 | 0.3904 | -0.1502 | -0.3900 | 0.9899 |
| Ferulic acid | -0.1712 | -0.2408 | -0.2082 | -0.1393 | -0.1786 | 1.7120 | -0.5953 | -0.1127 | -0.1041 | 0.0381 | 1.1557 |
| Gallic acid | -1.4801 | -0.0526 | -0.1127 | -0.2863 | -0.4181 | -1.6297 | 1.0324 | 1.2898 | 1.3643 | 0.2930 | 0.9434 |
| Protocatechuic acid | 1.6987 | 0.5415 | 0.3095 | 0.0632 | -0.4564 | 0.4136 | -2.1954 | -0.1002 | -0.4678 | 0.1933 | 0.9552 |
| Pyrocatechol | -1.3213 | 0.4216 | 0.6421 | 0.9658 | 0.9625 | -0.4310 | -2.2041 | 0.1938 | 0.0861 | 0.6852 | 1.0585 |
| Sinapyl alcohol | 0.1962 | 0.3646 | 0.4610 | 0.9423 | 0.8125 | -2.4695 | -1.1680 | 0.1582 | 0.1814 | 0.5214 | 0.8942 |
| Sinapyl aldehyde | -0.0408 | 0.0896 | -0.1630 | -0.3800 | -0.7194 | 1.6203 | -0.4317 | 0.0487 | 0.0516 | -0.0753 | 1.1664 |
| Syringaldehyde | 1.6663 | 0.0150 | -0.1850 | -0.4690 | 0.9904 | 2.1141 | -0.6483 | -0.2354 | -0.7152 | -0.5520 | 1.0234 |
| Syringic acid | 0.8156 | -0.6477 | -0.5094 | -0.2545 | -0.5781 | -2.1378 | 0.5958 | 0.7862 | 1.5095 | 0.4204 | 0.7819 |
| Sinapic acid | 2.5312 | -0.0691 | -0.1202 | -0.3305 | -0.8264 | 0.5454 | -0.6607 | -0.3148 | -0.4234 | -0.3314 | 0.8942 |
| Vanillic acid | -1.3275 | 0.8606 | 0.6540 | 1.4702 | 1.1337 | -1.4500 | -0.8313 | 0.2610 | -0.7373 | -0.0334 | 0.9919 |
| Vanillin | -0.5209 | -0.6409 | 0.3279 | 1.1635 | 1.0646 | -1.6524 | -1.4993 | 0.2245 | 0.4388 | 1.0943 | 0.9230 |
